# Drp1 modulates mitochondrial stress responses to mitotic arrest

**DOI:** 10.1038/s41418-020-0527-y

**Published:** 2020-03-19

**Authors:** Aida Peña-Blanco, Manuel D. Haschka, Andreas Jenner, Theresia Zuleger, Tassula Proikas-Cezanne, Andreas Villunger, Ana J. García-Sáez

**Affiliations:** 1 Interfaculty Institute of Biochemistry, Eberhard Karls University Tübingen, Tübingen, Germany; 2 Division of Developmental Immunology, Biocenter, Medical University of Innsbruck, Innsbruck, Austria; 3 Department of Molecular Biology, Interfaculty Institute of Cell Biology, Eberhard Karls University Tübingen, Tübingen, Germany; 4 Ludwig Boltzmann Institute for Rare and Undiagnosed Diseases, 1090 Vienna, Austria; 5 CeMM Research Center for Molecular Medicine of the Austrian Academy of Sciences, 1090 Vienna, Austria; 6 Present Address: Institute of Genetics, CECAD, University of Cologne, Cologne, Germany

**Keywords:** Cell biology, Cell death and immune response

## Abstract

Antimitotic drugs are extensively used in the clinics to treat different types of cancer. They can retain cells in a prolonged mitotic arrest imposing two major fates, mitotic slippage, or mitotic cell death. While the former is molecularly well characterized, the mechanisms that control mitotic cell death remain poorly understood. Here, we performed quantitative proteomics of HeLa cells under mitotic arrest induced with paclitaxel, a microtubule-stabilizer drug, to identify regulators of such cell fate decision. We identified alterations in several apoptosis-related proteins, among which the mitochondrial fission protein Drp1 presented increased levels. We found that Drp1 depletion during prolonged mitotic arrest led to strong mitochondrial depolarization and faster mitotic cell death as well as enhanced mitophagy, a mechanism to remove damaged mitochondria. Our findings support a new role of Drp1 in orchestrating the cellular stress responses during mitosis, where mitochondrial function and distribution into the daughter cells need to be coordinated with cell fate. This novel function of Drp1 in the cell cycle becomes best visible under conditions of prolonged mitotic arrest.

## Introduction

Antimitotic drugs that target microtubules, including taxol (generic name paclitaxel (PTX)) and vinca alkaloids, are extensively used in chemotherapy for several types of cancer [1]. In this scenario, understanding the molecular mechanisms of action of these drugs is of interest for researchers in order to define better therapeutic strategies that improve tumor responses in patients and reduce associated side effects [2]. Taxanes or vinca alkaloids interfere with microtubule dynamics, which activates the spindle assembly checkpoint (SAC) in mitosis [3]. The SAC ensures that the sister chromatids of each chromosome are stably attached to both poles of the mitotic spindle through their kinetochores, certifying the fidelity of chromosomal segregation during cell division [4]. SAC activation inhibits the anaphase promoting complex/cyclosome, an E3 ubiquitin ligase responsible for Cyclin B degradation, which controls exit from mitosis [5, 6]. Consequently, persistent SAC activation due to mitotic spindle attachment failures arrests cells in mitosis for longer times due to the maintenance of high Cyclin B levels [4].

Following prolonged mitotic arrest, cells present two major fates: (i) cell death in mitosis, mediated by the activation of the intrinsic apoptotic pathway [7–10]; or (ii) mitotic slippage, in which cells return to interphase without completing cell division due to slow but continuous degradation of Cyclin B [11]. According to the competing network model by Taylor et al. [12, 13], both possibilities are driven from two seemingly independent signaling networks with different thresholds, so that the fate of each individual cell is determined by the threshold that is achieved first. This model thus explains the large intra- and interline cell fate heterogeneity in response to antimitotic drugs. In contrast, a more recent study suggests crosstalk between both molecular machineries, proposing that perturbing one of the fates has an impact on the dynamics of the other one [14].

In contrast to slippage, the molecular regulation of cell death during prolonged mitosis is still not fully understood [12]. Although it is mediated mostly by the intrinsic apoptotic pathway and controlled by the proteins of the Bcl-2 family [15], the exact mechanism governing its initiation and execution are still under investigation [16]. As transcription is largely repressed in mitosis and to shed light on how the cell death fate is determined in mitotically arrested cells at the protein level, we carried out a quantitative proteomics screening aimed at identifying alterations in the molecular components associated with apoptotic cell death after PTX treatment.

We detected Drp1, the dynamin-like GTPase responsible for mitochondrial division, enriched in our proteomic screening. Drp1 has been extensively implicated in the intrinsic apoptotic pathway during interphase [17, 18]. Although Drp1 is not essential for apoptosis, it has been shown to affect the kinetics and extent of the death process by a yet obscure mechanism [19, 20]. Moreover, Drp1 is regulated in a cell cycle dependent manner, showing an expression peak during mitosis when mitochondria get highly fragmented [21, 22]. Drp1 indeed plays a role in mitochondrial fragmentation during mitosis, which is thought to promote proper distribution of the mitochondrial material in the daughter cells [23]. In a previous study, Diaz-Martinez et al. proposed that Drp1 contributes to mitotic checkpoint adaptation after extended mitotic arrest in U2OS cells [14]. However, this work partially relied on the use of Mdivi-1, which has been since then discarded as a Drp1-specific inhibitor [24]. Hence, the role of Drp1 remained to be clarified by genetic means.

Here, we found that Drp1 depletion had a strong effect on mitochondrial polarization upon PTX treatment and compromised the duration of mitotic arrest by accelerating cell death. Interestingly, besides enhancing the loss of mitochondrial membrane potential, Drp1 depletion also increased mitophagy during prolonged mitotic arrest. These results reveal a more complex scenario than expected, where Drp1 exerts a coordinating function at the intersection between mitotic cell death and mitophagy in response to cellular stress induced by prolonged mitotic arrest.

## Results

### Paclitaxel-treated HeLa cells present common apoptotic hallmarks

**Fig. 1:**
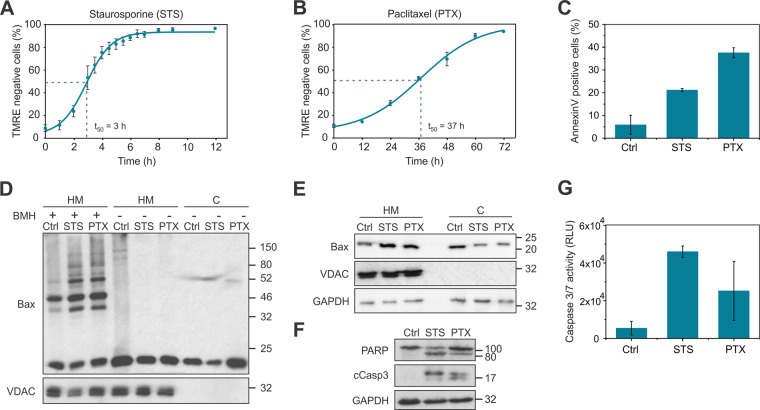
Paclitaxel-treated HeLa cells present common hallmarks of apoptosis. **a**, **b** TMRE kinetics of HeLa cells during staurosporine (STS) or paclitaxel (PTX) treatment. The values represent the mean of at least three independent experiments. Lines correspond to the best data fit. Error bars correspond to standard deviation. **c** Annexin V-FITC staining of HeLa cells after treatment with DMSO (Ctrl), STS (3 h), and PTX (37 h). Error bars correspond to standard deviation. **d** Bax oligomerization in HeLa cells after treatment with DMSO (Ctrl), STS (3 h), or PTX (37 h). Cells were separated in heavy membrane (HM) and cytosolic (C) fractions. Fractions were incubated with the crosslinker bismaleimidohexane (BMH). VDAC1 was used as a mitochondrial marker. Representative immunoblot of three independent experiments. **e** Bax subcellular localization in HM and cytosolic (C) fractions after treatment with DMSO (Ctrl), STS (3 h), or PTX (37 h). VDAC and GAPDH were used as mitochondrial and cytosolic marker, respectively. Representative immunoblot of three independent experiments. **f** PARP and cleaved caspase-3 (cCasp3) immunoblot of HeLa cells after treatment with DMSO (Ctrl), STS (3 h), or PTX (37 h). Representative immunoblot of three independent experiments. **g** Caspase-3/7 activity as determined by luminescence in HeLa cells after treatment with DMSO (Ctrl), STS (3 h), or PTX (37 h). Error bars correspond to standard deviation.

To identify proteins involved in the initiation of mitotic cell death, we chose HeLa cells as a cell model, since this cell line mostly undergoes apoptosis upon extended mitotic arrest [7]. Considering that apoptosis is a dynamic process and to set the optimal time point to study proteomic changes associated with mitotic cell death, we monitored mitochondrial depolarization as a probe for mitochondrial outer membrane (MOM) permeabilization. We stained HeLa cells with the mitochondrial potential (Ψ_m_) dependent dye TMRE and induced cell death by treatment with PTX, a microtubule-stabilizer drug which triggers extended mitotic arrest by activating the SAC [25]. As a reference for apoptosis, we also induced cell death with the commonly used drug staurosporine (STS), a pan-kinase inhibitor. We monitored the levels of TMRE fluorescence at different time after treatment using flow cytometry and determined *t*_50_ as the time point when cells presented 50% of TMRE fluorescence intensity (Fig. 1a, b). For PTX- and STS-treated HeLa cells, *t*_50_ corresponded to 37 h and 3 h, respectively. Given that dead cells, which have low TMRE intensity, present high caspase activity that could affect the proteome, we used the value *t*_50_ as the optimal time for analyzing proteomic changes involved in mitotic cell death decision-making.

To characterize the features of cell death at time *t*_50_, we assessed several apoptotic markers, such as phosphatidylserine (PS) exposure at the plasma membrane, Bax oligomerization, and subcellular localization, as well as effector caspase activation. Cells treated with PTX for 37 h showed around 40% of Annexin V-positive cells, indicative of PS exposure (Fig. 1c). Under these conditions, the proapoptotic protein Bax was detected as higher-order oligomers (Fig. 1d) and recruited to the heavy membrane (HM) fraction containing mitochondria (Fig. 1e) in a similar extent to STS-treated cells, showing activation of the intrinsic apoptotic pathway. Furthermore, we detected by immunoblot the cleavage of the caspase substrate PARP1 along with the cleavage of caspase-3 (Fig. 1f). To confirm the activation of effector caspases, we measured their activity using a luminescence assay and detected caspases-3/7 activation upon PTX treatment, although to a lower extent than with STS treatment (Fig. 1g). Our results indicated that common apoptotic hallmarks are well detectable after 37 h of PTX treatment, setting a suitable time point to study proteomic changes during mitotic cell death.

### Drp1 levels are increased in HeLa cells treated with paclitaxel

**Fig. 2:**
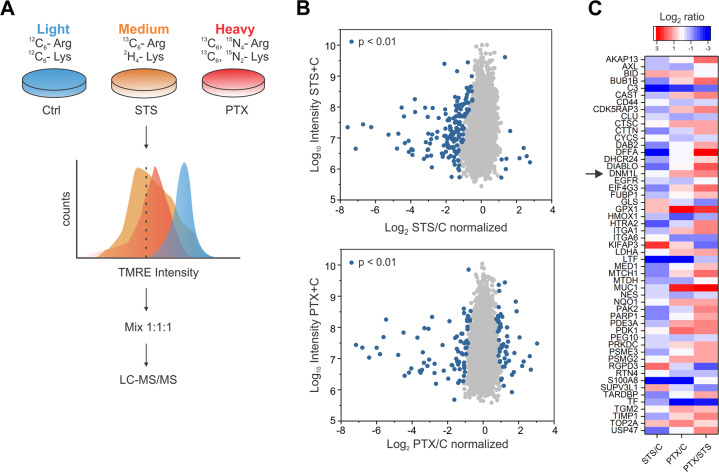
Drp1 levels are increased in HeLa cells treated with paclitaxel. **a** Design of three-state stable isotope labeling by amino acids in cell culture (SILAC) experiment. HeLa cells were cultured in media with medium (3 h STS) or heavy (37 h PTX) isotopes of arginine and lysine. Control cells without treatment were cultured in light media. Cells were stained with TMRE and sorted using FACS. Cells were lysed, mixed in a ratio 1:1:1 by protein mass and analyzed by mass spectrometry. **b** Scatter plot showing Log_2_ (STS/C) vs. Log_10_ Intensity (STS + C) (top plot) or Log_2_ (PTX/C) vs. Log_10_ Intensity (PTX + C) (bottom plot). Significantly changed proteins are shown in blue (*p* < 0.01). **c** Heat map of proteins involved in apoptosis according to the GOBP annotation. Rows correspond to the proteins and columns to the conditions of the SILAC experiment. Drp1 (DNM1L) is highlighted with an arrow.

We then performed a three-state SILAC analysis according to the experimental design in Fig. 2a. The experiment consisted of three samples: HeLa cells cultured with heavy isotope-labeled amino acids (Heavy) and treated with PTX for 37 h; HeLa cells cultured with medium isotope-labeled amino acids (Medium) and treated with STS for 3 h; and control HeLa cells cultured in light amino acids (Light) without treatment. In order to discard dead cells, we performed FACS after TMRE staining and excluded cells that presented TMRE intensity lower than 50%. This way we ensured that only cells with weak mitochondrial depolarization were considered in our analysis. After cell sorting, the lysates of heavy, medium, and light samples were mixed in a ratio 1:1:1 and analyzed by liquid chromatography-tandem MS (LC-MS/MS).

From the MS experiment, we obtained the SILAC ratios, which allowed us to quantify the proteomic changes between samples. In particular, we focused our analysis on the modifications between treated and control samples. We normalized Log_2_ STS/control (STS/C) and normalized Log_2_ PTX/control (PTX/C) to identify proteins that were significantly changed upon each of the treatments. Figure 2b shows the protein SILAC ratios plotted against the Log_10_ intensities, which identified a population of proteins that were changed after treatment with *p* < 0.01. This analysis yielded a list of 161 and 131 significantly changed proteins for STS and PTX treatment, respectively.

Once the significant changes in the proteomes were defined, we classified the proteins involved in cell death using the Gene Ontology biological process (GOBP) annotation [26]. We plotted the proteins engaged in cell death in a heat map, which was color-coded based on the Log_2_ ratios (Fig. 2c). As expected, in the STS/C condition we could detect enrichment of the BH3-only protein Bid [27] as well as a negative ratio for USP47, a regulator of cell survival [28]. Regarding the PTX/C condition, several proteins that have a role in cell cycle arrest or mitotic checkpoint regulation appeared in our list, such as BUB1B and CDK5RAP3 [29, 30]. Drp1 levels also appeared upregulated under PTX treatment, but not upon apoptosis induction with STS. We validated by western blot that Drp1, specifically in its phosphorylated form, was accumulated during mitotic arrest (Fig. S1). Interestingly, Drp1 has been proposed to regulate Bcl-2 proteins and to affect apoptosis [16, 17]. Since Bcl-2 proteins drive cell death responses also in mitotic cell death [9], we decided to investigate the role of this protein in more detail.

### Mitochondrial depolarization is enhanced during PTX-induced mitotic arrest in Drp1-depleted cells

**Fig. 3:**
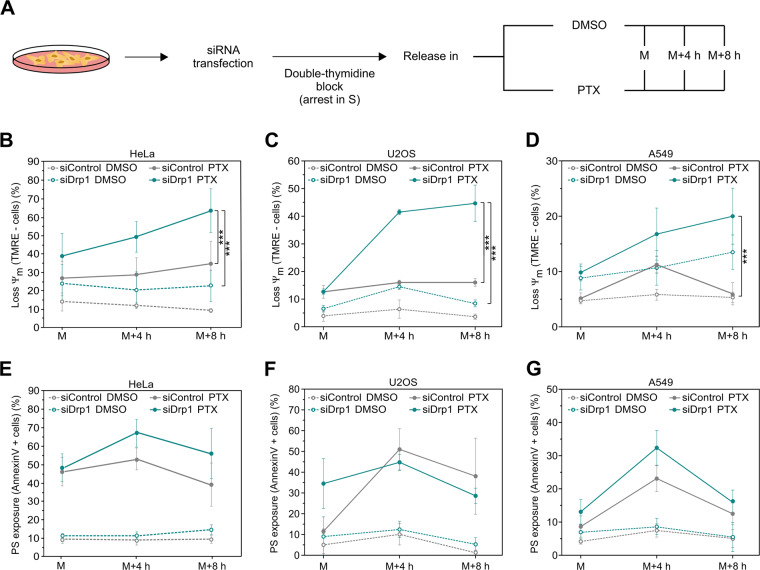
Drp1 is required to preserve mitochondrial membrane potential during PTX-induced mitotic arrest. **a** Scheme of the cell cycle synchronization experiment. Drp1 was downregulated in HeLa cells by siRNA transfection. HeLa cells were synchronized at the G1/S boundary using a double-thymidine block. Afterwards cells were released back into the cell cycle in media with DMSO or PTX and harvested at several time points to study extended mitotic arrest: 12 h (M), 16 h (M + 4 h), and 20 h (M + 8 h). **b** Quantification of the percentage of cells with Ψ_m_ loss in siControl (gray) and siDrp1 (blue) HeLa cells released into media with PTX. Mitotic cells were harvested after 12 h (M), 16 h (M + 4 h), and 20 h (M + 8 h) by mitotic shake-off. DMSO-released cells (empty circles) are shown as a control. The values represent the mean and the SD of four independent experiments. ***p < 0.001 using Student’s *t* test. **c** Same as in (**b**) but in siControl (gray) and siDrp1 (blue) U2OS cells released into media with PTX. ***p < 0.001 using Student’s *t* test. **d** Same as in (**b**) but in siControl (gray) and siDrp1 (blue) A549 cells released into media with PTX. ***p < 0.001 using Student’s *t* test. **e** Quantification of the percentage of cells with PS exposure at the plasma membrane in siControl (gray) and siDrp1 (blue) HeLa cells released into media with PTX. Mitotic cells were harvested after 12 h (M), 16 h (M + 4 h), and 20 h (M + 8 h) by mitotic shake-off. DMSO-released cells (empty circles) are shown as a control. The values represent the mean and the SD of four independent experiments. ***p < 0.001 using Student’s *t* test. **f** Same as in (**e**) but in siControl (gray) and siDrp1 (blue) U2OS cells released into media with PTX. ***p < 0.001 using Student’s *t* test. **g** Same as in (**e**) but in siControl (gray) and siDrp1 (blue) A549 cells released into media with PTX. ***p < 0.001 using Student’s *t* test. All *t*-tests were two-sided.

To examine if there is indeed an effect of Drp1 on mitotic cell death and/or mitotic arrest, we performed an RNA interference-mediated knockdown of Drp1 (siDrp1) followed by a double-thymidine arrest to synchronize HeLa cells at the G1/S boundary. We released cell cycle-arrested cells at G1/S transition in DMSO or PTX to impose mitotic arrest for different amounts of time: 12 h (M), 16 h (M + 4 h), and 20 h (M + 8 h) (Fig. 3a). We confirmed the depletion of Drp1 by immunoblot and confocal microscopy (Fig. S2A, B). Drp1 levels dropped upon siRNA treatment and this was not affected by the presence of PTX or DMSO (Fig. S2A). In addition, mitotic cells transfected with siDrp1 showed hyperfused mitochondria (Fig. S2B), in agreement with the downregulation of Drp1 in our system.

We then quantified the percentage of mitotic cells using flow cytometry by harvesting the ‘M’, ‘M + 4 h’ and ‘M + 8 h’ populations followed by immunostaining of phosphorylated Ser10 Histone-3 as a marker for mitosis. Depletion of Drp1 did not have any impact on the percentage of cells undergoing extended mitotic arrest (Fig. S3A), indicating that Drp1 has no role in mitotic entry or in the extension of the mitotic arrest in HeLa cells during the amount of time we measured.

Next, we tested whether Drp1 could affect mitotic cell death upon prolonged arrest. To this end, we first quantified the mitochondrial membrane potential in mitotically arrested cells treated with control siRNA or siRNA targeting Drp1 by flow cytometry. Mitochondrial depolarization occurred in siDrp1 cells in a treatment and time-dependent manner (Fig. 3b). To support our observations in HeLa cells, we employed two additional cell lines, U2OS and A549, that mainly undergo mitotic slippage in the presence of PTX [7]. We validated Drp1 downregulation in these cell lines during mitotic arrest by immunoblot (Fig. S2C). In U2OS and A549 cells, mitochondrial depolarization in the siDrp1 conditions with PTX was also enhanced (Fig. 3c, d). Altogether, Drp1 depletion had a strong effect on mitochondrial depolarization during PTX-induced extended mitotic arrest across cell lines but had no detectable effect on normal cell cycle progression over the time period analyzed.

Of note, siDrp1 HeLa cells released in DMSO consistently presented lower mitochondrial potential than cells treated with scramble siRNA, indicating a certain level of mitochondrial dysfunction in Drp1-depleted HeLa cells [31]. We could also observe that Drp1 downregulation in A549 cells was sufficient to depolarize mitochondria in the presence of DMSO to a similar extent than upon PTX treatment, suggesting again that Drp1 contributes to mitochondrial function in steady state, likely via its role in mitochondrial dynamics.

In addition, we measured PS exposure at the outer leaflet of the plasma membrane as another cell death marker using Annexin V staining. As expected, staining with Annexin V-FITC showed a higher population of dying cells upon prolonged PTX treatment compared with cells released in DMSO. Only minor differences between Drp1-depleted cells and control cells were observed in all three cell lines tested (Fig. 3e–g), suggesting that Drp1 plays more critical roles in the maintenance of mitochondrial membrane potential but does not directly impact the dynamics of PS exposure and, hence, caspase activation. Similar results were obtained with a colony formation assay (Fig. S4). Of note, as an additional control for the specificity of the effects seen by Drp1 depletion, we corroborated our findings using an alternative siRNA targeting Drp1. Moreover, application of Mdivi-1, which, despite not being highly selective, prevents mitochondrial fission, gave similar results (Fig. S5).

### Drp1 function on mitochondrial depolarization during mitotic arrest is independent of microtubule dynamics

Alternative splicing of Drp1 generates functionally distinct isoforms. Drp1 isoforms that exclude the second alternative exon localize to microtubule bundles in a process regulated by CDK-mediated phosphorylation [32]. Given that HeLa cells contain 40–60% of these isoforms [32] and that our PTX treatment stabilizes microtubules, we controlled for the possibility that the Drp1 function during mitotic arrest in our experimental conditions was dependent on the role of Drp1 on stabilized microtubules rather than on its action on mitochondria.

**Fig. 4:**
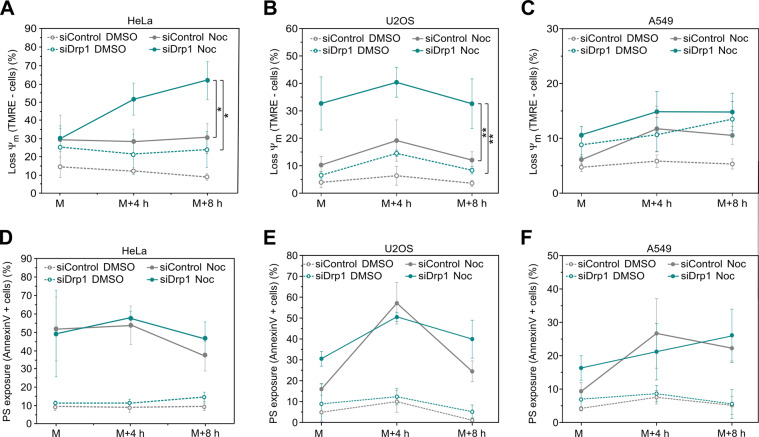
Drp1 function on mitochondria depolarization during mitotic arrest is independent on microtubules assembly. **a** Quantification of the percentage of cells with Ψ_m_ loss in siControl (gray) and siDrp1 (blue) HeLa cells released into media with Noc. Mitotic cells were harvested after 12 h (M), 16 h (M + 4 h), and 20 h (M + 8 h) by mitotic shake-off. DMSO-released cells (empty circles) are shown as a control. The values represent the mean and the SD of four independent experiments. *p < 0.05 using two-sided Student’s *t* test. **b** Same as in (**b**) but in siControl (gray) and siDrp1 (blue) U2OS cells released into media with Noc. **p < 0.01 using two-sided Student’s *t* test. **c** Same as in (**a**) but in siControl (gray) and siDrp1 (blue) A549 cells released into media with Noc. **d** Quantification of the percentage of cells with PS exposure at the plasma membrane in siControl (gray) and siDrp1 (blue) HeLa cells released into media with Noc. Mitotic cells were harvested after 12 h (M), 16 h (M + 4 h), and 20 h (M + 8 h) by mitotic shake-off. DMSO-released cells (empty circles) are shown as a control. The values represent the mean and the SD of four independent experiments. **e** Same as in (**d**) but in siControl (gray) and siDrp1 (blue) U2OS cells released into media with Noc. **f** Same as in (**d**) but in siControl (gray) and siDrp1 (blue) A549 cells released into media with Noc.

To this aim, we employed the drug nocodazole (Noc), which also promotes mitotic arrest but in this case it induces microtubule depolymerization instead of stabilizing them like PTX [33]. We performed a double-thymidine arrest followed by release in DMSO or Noc in HeLa, U2OS, and A549 cells and assessed the levels of mitotic cells, mitochondrial membrane depolarization and PS exposure at the plasma membrane at several time points after release. Similar to PTX, Noc treatment triggered mitotic arrest with similar efficiency (Fig. S3B). Cells arrested in mitosis by Noc treatment presented again higher mitochondrial depolarization rates in the absence of Drp1 (Fig. 4a–c), as well as a modest increase in the number of Annexin V-positive cells during prolonged mitotic arrest (Fig. 4d–f). These findings indicate that the role of Drp1 during extended mitotic arrest is independent of microtubule dynamics.

### Drp1 depletion compromises mitotic arrest by accelerating cell death in mitosis

Using Annexin V staining in bulk analyses appeared less suited to assess the impact of Drp1 depletion on cell death per se. Hence, to further characterize the role of Drp1 in cell fate decisions during extended mitotic arrest, we carried out live cell imaging to determine cell fate and mitotic durations at the single-cell level upon treatment. We released the G1/S-arrested cells in media containing DMSO, PTX, or Noc and visualized them from the time of nuclear envelope breakdown till either onset of anaphase, mitotic cell death, or mitotic slippage. Representative images are shown in Fig. S6. This allowed us to determine the durations from mitotic arrest to death or mitotic exit for each cell.

**Fig. 5:**
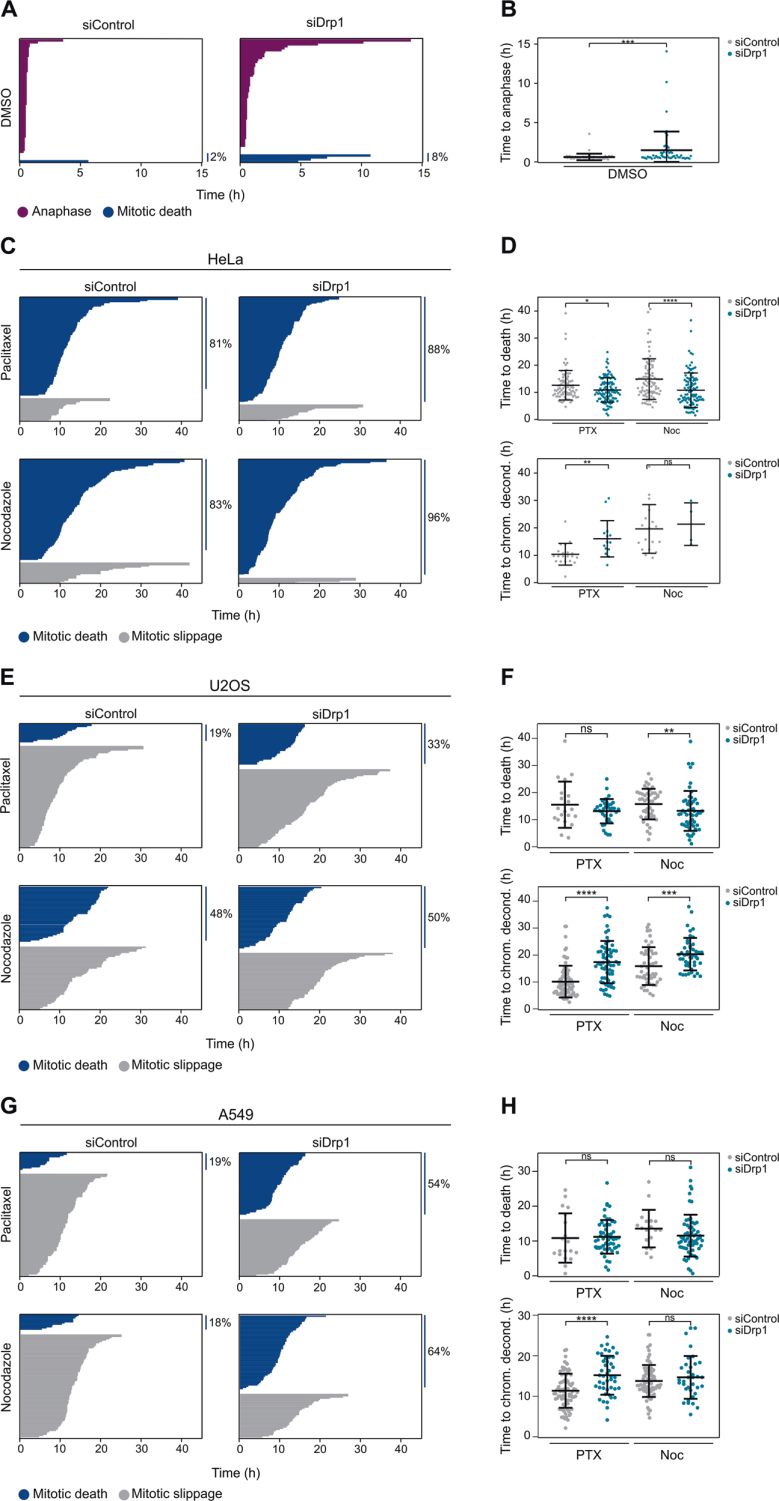
Drp1 depletion compromises mitotic arrest by accelerating cell death in mitosis. **a** Fate profiles of single thymidine-arrested siControl or siDrp1 HeLa cells released into DMSO. Each bar represents the duration of mitosis of one cell. Bar color indicates cell fate. Percentage of cells undergoing mitotic cell death is indicated. At least 50 cells were quantified in each condition. **b** Box (interquartile range) and whisker (min to max) plots showing the time to anaphase for individual cells after treatment. ***p < 0.001 using Mann–Whitney test. **c** Fate profiles of single thymidine-arrested siControl or siDrp1 HeLa cells released into PTX or Noc. Each bar represents the duration of mitotic arrest of one cell. Bar color indicates cell fate. Percentage of cells undergoing mitotic cell death is indicated. At least 50 cells were quantified in each condition. **d** Box (interquartile range) and whisker (min to max) plots showing the time between mitotic entry and mitotic death or slippage for HeLa cells monitored in (**c**). ****p < 0.0001, **p < 0.001, *p < 0.05, ns no significant using Mann–Whitney test. **e** Fate profiles of single thymidine-arrested siControl or siDrp1U2OS cells released into PTX or Noc. Each bar represents the duration of mitotic arrest of one cell. Bar color indicates cell fate. Percentage of cells undergoing mitotic cell death is indicated. At least 50 cells were quantified in each condition. **f** Box (interquartile range) and whisker (min to max) plots showing the time between mitotic entry and mitotic death or slippage for U2OS cells monitored in (**e**). ***p < 0.0001, **p < 0.001, *p < 0.05, ns no significant using Mann–Whitney test.

Of note, the release of cells into DMSO modestly extended mitotic durations in siDrp1 HeLa cells that progressed to anaphase (Fig. 5a, b), in agreement with a role of Drp1 in preserving mitochondrial dynamics and hence function, which play a role in cell cycle progression [34].

PTX and Noc treatment led to mitotic cell death of the majority of HeLa cells and only a small fraction escaped by mitotic slippage (Fig. 5c) supporting the tendency of this cell line to die in mitosis upon prolonged mitotic arrest. Interestingly, Drp1-depleted cells displayed even higher amounts of dead cells in mitosis upon either treatment (Fig. 5c), which was also characterized by shorter time to death compared with control siRNA cells (Fig. 5d, top). The few cells that underwent mitotic slippage appeared arrested in mitosis for longer periods of time when pretreated with siDrp1 (Fig. 5d, bottom), although the low number of cells that could be analyzed precluded a clear conclusion on Drp1’s impact on mitotic slippage.

U2OS and A549 cells are more slippage prone during extended mitotic arrest [7, 9], consistent with our observations in single-cell fate experiments (Fig. 5e, g). Drp1 downregulation clearly enhanced the percentage of cells dying in mitosis, or, at a minimum, the time to death upon treatment with PTX or Noc in U2OS (Fig. 5e, g) as well as A549 cells (Fig. 5f, h, top panels). Of note, depletion of Drp1 in U2OS and A549 cells extended the time required for chromatin decondensation in cells undergoing mitotic slippage (Fig. 5f, h, bottom panels), corroborating the relevance of Drp1 in mitotic progression and our findings obtained in HeLa cells.

To further support the increase in apoptotic cells upon Drp1-depeletion, we additionally analyzed PARP1 cleavage, as well as the levels of some Bcl-2 proteins family members known to control mitotic cell death [9] by immunoblot (Fig. S7). PARP cleavage was detected earlier in siDrp1-depleted cells arrested in mitosis, indicating faster apoptotic kinetics in the absence of Drp1. In addition, an accumulation of the proapoptotic BH3-only protein NOXA correlated with a shift toward mitotic cell death in the absence of Drp1 (Fig. S7).

Taken together, these results show that Drp1 is required for timed mitotic exit under normal conditions but also affects the rate of mitotic slippage in the presence of microtubule targeting agents. Moreover, mitotic cell death shows higher penetrance and faster kinetics when Drp1 is lacking. Our findings suggest a role for the functional state of mitochondria controlled by Drp1 in determining cell fate in mitosis, which is exacerbated and best highlighted during extended mitotic arrest.

Since Drp1 depletion results in hyperfused mitochondria, it remained possible that the effects observed were due to alterations in mitochondrial homeostasis in general and therefore not specific to Drp1. To disentangle mitochondrial hyperfusion from this potentially new Drp1 function, we analyzed cell death and mitochondrial potential during mitotic arrest under conditions of MFN2 overexpression in synchronized HeLa cells. MFN2 overexpression resulted in elongated mitochondria, as expected, but did not recapitulate the same phenotype as Drp1 depletion (Fig. S8). In agreement with this, co-depletion of Drp1 and MFN1/2, aimed at reducing the mitochondrial hyperfusion, still retained the same phenotype as Drp1 knockdown (Fig. S8), supporting a specific function of Drp1 that is independent of its role on mitochondrial dynamics.

### Loss of Drp1 increases mitophagy in HeLa cells during mitotic arrest

In our system, the increase of mitochondrial depolarization in siDrp1-treated cells during mitotic arrest only correlated with a subtle increase in PS exposure at the plasma membrane. This suggested that the loss of mitochondrial membrane potential detected during PTX/Noc-induced mitotic arrest was not solely a consequence of MOM permeabilization and caspase activation, but related to other cellular processes affecting the functional state of mitochondria. Mitochondrial membrane potential loss is a well-known trigger of mitophagy [35], a selective form of autophagy that targets dysfunctional mitochondrial to autophagosomes for degradation. Hence, we wondered whether Drp1 played a role in mitophagy during extended mitotic arrest.

**Fig. 6:**
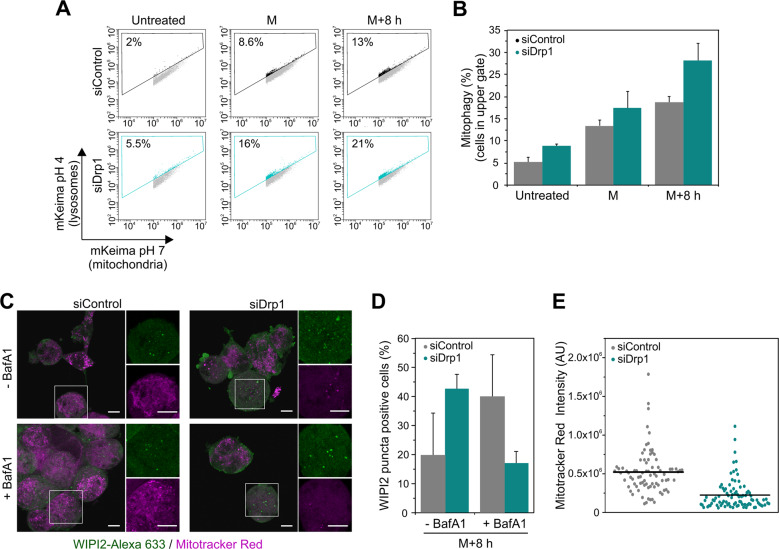
Mitophagy is enhanced in Drp1-depleted cells during mitotic arrest. **a** Mito-mKeima-Red dot plots of siControl (top panels) and siDrp1 (bottom panels) HeLa cells of untreated or cell cycle-arrested cells released in PTX and harvested at M (12 h) or M + 8 h (20 h). Dot plots represent the fluorescence emission of mito-Keima at the mitochondria (*x* axis) vs. its emission at the lysosomes (*y* axis) due to the different pH of both organelles. The percentage of cells within the box indicates the rate of mitophagy. **b** Percentage of mitophagy as displayed in (**a**). The values represent the mean and the SD of three independent experiments. **c** siControl (left) and siDrp1 (right) ‘M + 8 h’ HeLa cells were stained with MitoTracker Red CMXRos (magenta) for 1 h. Cells were fixed and immunostained with anti-WIPI2-Alexa Fluor 633 (green) and visualized by confocal microscope. Scale bar, 10 µm. **d** Quantification of mitotic cells with WIPI2 puncta in siControl and siDrp1 ‘M + 8 h’ HeLa cells as performed in (**c**). At least 25 cells were quantified per condition in each repetition. Data represent the mean and SD of three independent experiments. **e** Quantification of Mitotracker Red CMXRos intensity in siControl and siDrp1 ‘M + 8 h’ mitotic HeLa cells as performed in (**c**). At least 25 cells were quantified per condition in each repetition. Data represent the mean (line) and the individual intensity value for each cell (dots).

To test this hypothesis, we measured mitophagy levels under siDrp1 conditions using the mKeima-Red-mito vector [36, 37]. This vector contains fused GFP and mCherry targeted to mitochondria and is sensitive to pH changes: for cytosolic mitochondria (pH 7), mKeima emits in both the GFP and Cherry channels and results in the yellow signal. In contrast, for mitochondria inside lysosomes (pH 4), the GFP fluorescence is quenched and mKeima emits only in the mCherry channel (red signal). We transfected cells with mKeima-Red-mito and measured the changes in the ratio of mKeima emission in the green and the red channels at different time points during mitotic arrest after cell cycle synchronization and release into PTX using flow cytometry (Fig. 6a, b). Strikingly, we observed a clear induction of mitophagy during mitotic arrest with higher rates detectable in siDrp1-treated HeLa cells.

To investigate whether autophagy was activated by default during mitotic arrest in a Drp1-dependent manner, we imaged endogenous WIPI2 stained by immunofluorescence during mitotic arrest, together with MitoTracker Red CMXRos, a mitochondrial potential dependent dye. Binding of WIPI2 to PtdIns3P at nascent autophagosomes is demonstrated by the appearance of subcellular puncta [38, 39], which can be used as a marker of autophagosomes formation. The number of cells displaying WIPI2 puncta was higher in mitotic siDrp1 ‘M + 8 h’ HeLa cells (cells with extended mitotic arrest) compared with siControl cells (Fig. 6c, d). This shows that the number of autophagosomes increases in the absence of Drp1 during mitotic arrest. In agreement with our flow cytometry results, siDrp1 cells presented lower mitochondrial potential (Fig. 6e).

## Discussion

While it is well accepted that the intrinsic apoptotic pathway controls cell death during extended mitotic arrest [7, 9, 15, 16], the exact molecular mechanisms that initiate these cell death decisions are not well understood. Here, we identified the mitochondrial protein Drp1 as a novel player in the regulation of mitotic cell death by using quantitative proteomics in HeLa cells treated with PTX. Our findings reveal a modulatory function for Drp1 in normal mitotic progression in steady state as well as upon extended mitotic arrest, where it plays an orchestrating role at the interface of the cellular pro-survival vs. pro-death responses.

We found that Drp1 levels were significantly increased during cell death in HeLa cells treated with PTX. Drp1 has been proposed as a player in apoptosis, given that it colocalizes with Bax, required for MOM permeabilization, at discrete foci [40] and that its absence delays cytochrome c release [41, 42]. However, Drp1 function in apoptosis is controversial, since other studies have indicated that Drp1 has no or little impact on the kinetics of apoptosis [43, 44]. On the other hand, mitochondria get drastically fragmented in a Drp1-dependent manner during mitosis in order to ensure proper organelle segregation [23]. At this stage, Drp1 function is regulated by phosphorylation via CDK1/Cyclin B, mitotic kinases like RALA and RALBP1, and other posttranslational modifications, such as SUMOylation [23, 45, 46]. Phosphorylation of Bid during mitosis also plays a role in sensitizing cells to apoptosis during delayed mitotic exit [15]. It is thus likely that the Drp1 enrichment and phosphorylation that we detected upon PTX treatment resulted from normal stabilization of Drp1 protein during mitosis [21]. Our findings bring together the documented role of Drp1 in mitosis and its proposed role in apoptosis by supporting a model in which Drp1 functions as a regulator of mitochondria quality control and stress responses needed for balanced organelle segregation into the daughter cells during mitosis.

Indeed, our data show that Drp1 participates in mitotic cell death regulation. The absence of Drp1 increased the penetrance and kinetics of death in mitosis, which argues against a pro-death function of Drp1 during prolonged mitotic arrest. Yet, Drp1 has been recently proposed to play a proactive role in mitotic slippage, albeit mechanistically ill-defined [14, 47]. Consistently, we noted prolonged time to chromatin decondensation upon Drp1 siRNA in steady state as well as under conditions of mitotic arrest. However, altogether our data rather support the possibility that the effects observed using Drp1 knockdown are not related to a direct function of Drp1 in mitotic slippage but a consequence of altered mitochondrial shape and function due to reduced organelle fission during mitotic progression. In support of this notion, in cells without Drp1 we also observed an increase of mitotic cell death, as well as longer mitotic duration for cells that reenter the cell cycle even in the absence of antimitotic drugs.

Our results and those from others [48, 49] indicate that Drp1 depletion impaired normal cell cycle progression and that proper mitochondrial dynamics are crucial for timed mitotic exit during normal cell division and upon extended mitosis. The comparable effects between Noc and PTX treatments that we observed indicate that this function is independent of microtubules assembly or structure. Our findings were consistent in cell lines with opposite fates upon extended mitotic arrest. While HeLa cells tend to undergo mitotic cell death, U2OS and A549 cells are slippage prone [7], reinforcing our model of Drp1 as general key player in determining cell cycle progression during prolonged mitosis.

We found that the loss of mitochondrial potential induced by Drp1 depletion, exacerbated during extended mitotic arrest, did not fully correlate with apoptosis induction, as reported by PS exposure. These findings suggest a role for Drp1 in preserving mitochondria bioenergetics, which likely becomes more relevant during prolonged mitotic arrest. In fact, mitosis is a highly energy-demanding process [50] and the loss of mitochondrial function has been observed in cells arrested for long periods of times in mitosis [10]. In this scenario, it seems reasonable that impairment of mitochondrial dynamics during mitotic arrest causes insufficient mitochondrial respiration to ensure proper and timely mitotic exit, thereby explaining the adverse effect of Drp1 knockdown promoting mitotic cell death.

In line with the loss of mitochondrial function mentioned above, we also detected an increased rate of autophagy, and specifically mitophagy, upon extended mitotic arrest that was enhanced under conditions of Drp1 depletion. Mitophagy is responsible for clearing defective mitochondria and several studies support however a role for Drp1 in the generation of mitochondrial fragments of a suitable size for autophagosome engulfment [51–53]. Under our experimental settings, we found that the role of Drp1 in maintaining a functional mitochondrial network dominated over its function in generating small mitochondrial fragments for autophagosome engulfment. In our study, increased rates of mitophagy induced by Drp1 loss during extended mitotic arrest unite the strong mitochondrial depolarization with the role for Drp1 in quality control. Such coordinating role at the intersection between mitochondrial function, quality control, and stress responses during extended mitosis positions Drp1 as a central player to secure proper segregation of fit mitochondria in cell division.

Worth mentioning here is that it has been recently reported that prolonged mitotic arrest in Cdc20 deficient MEF cells results in mitophagy, reduced ATP levels, and the activation of AMPK, contributing to cell death [10], a notion supported by our findings (Fig. 6b). However, in contrast to this study, we found that Drp1 loss during mitotic arrest further increased mitophagy and promoted mitotic cell death over slippage. These differences may be attributed to different effects of PTX treatment vs. Cdc20 depletion and/or to the use of different cellular systems. While the consequences of Cdc20 depletion were mostly studied in MEF, we interrogated three different human cancer cell lines, showing similar effects. Methodological differences in the quantification of mitophagy, mKeima staining, and flow cytometry vs. co-localization of LC3-GFP with Mitotracker by microscopy, which is limited by the number of cells that can be analyzed, may impact on the results obtained. Regardless, both PS exposure as well as changes in the ratio between cell death and slippage by single-cell live cell imaging clearly support an increase in mitotic cell death in Drp1-depleted cells in our study, which we think may be a more accurate estimation than the analysis of the time to death alone.

Overall, here we show that the mitochondrial fission protein Drp1 is a critical player for proper cell cycle progression by connecting the functional state of mitochondria with the cellular stress responses activated during mitotic progression. During prolonged mitotic arrest, the loss of mitochondrial potential promotes both mitophagy and cell death in mitosis, modulated by Drp1 function, thereby activating different mechanisms to avoid mitotic exit in the presence of damaged or insufficient numbers of mitochondria. We propose a new model where Drp1-regulated signaling would maintain mitochondrial function, secure mitotic fidelity, as well as adaptation and slippage. Unresolved mitochondrial stress, caused by impaired Drp1 function would tip the balance toward mitotic cell death by apoptosis, securing genomic stability. Since Drp1 expression correlates with the expression of mitotic transition genes in several tumors [54], our results pinpoint Drp1 as a potential target to improve treatment responses to antimitotic drugs and, potentially, tumor responsiveness to such drugs. Therefore, the results reported here could have implications in the exploitation of mitochondrial dynamics in combination with microtubule poisons in the clinics.

## Materials and methods

### Reagents

The reagents used in this study were as follows (working concentrations are specified): STS (1 µM, LC Laboratories), PTX (50 nM, Sigma-Aldrich), Noc (1 µM, Sigma-Aldrich) DMSO (Sigma), TMRE (100 nM, Life Technologies), Annexin V-FITC (BD Bioscience), propidium iodide (2.5 µg/mL, Sigma-Aldrich), MitoTracker Red CMXRos (100 nM, Thermo Fisher Scientific), DAPI (Thermo Fisher Scientific). Cells were HeLa Kyoto from EMBL, A549 ATCC® CCL-185, and U2OS ATCC® HTB-96.

### Cell culture

HeLa cells were cultured in low glucose Dulbecco’s modified Eagle’s medium (DMEM) (Sigma-Aldrich) with 10% fetal bovine serum (FBS), 1% penicillin–streptomycin (P/S) (Thermo Fisher Scientific) at 37 °C in a humidified incubator containing 5% CO_2_.

### siRNA transfection

siRNA transfection was performed in six-well plates. For each well, 5 nM of siRNA were premixed with 1 µL lipofectamine (Life Technologies) in 200 µl optimem (Thermo Fischer Scientific). The mixture was incubated for 20 min at room temperature before adding it into the wells containing 1 mL optimem. After 6 h, 1 mL of DMEM supplemented with 20% FBS and 1% P/S was added to each well. siRNA transfection was performed 36 h before the start of double-thymidine block and 4 days before harvesting/fixing the cells for flow cytometry or microscopy experiments. The levels of the endogenous protein were checked by immunoblot at different moments during the procedure. The following siRNA were used: siDrp1 (GGAGCCAGCUAGAUAUUAA) and siControl (D-001810-01-05) from Dharmacon.

### Cell cycle synchronization

Synchronization of HeLa cells was either done by a single 2 mM thymidine (Sigma-Aldrich) arrest for 24 h (Figs. 5 and 6c, d) or a double-thymidine arrest consisting in 22 h arrest followed by 9 h release into DMEM followed by 17 h arrest (Figs. 3, 4, 6a, b). After arrest, cells were released into media containing the following drugs: 50 nM PTX (Sigma-Aldrich), 1 µM Noc (Sigma-Aldrich), or 100 nM BI2536 (Selleck). Dimethyl sulfoxide (Sigma-Aldrich) was used as solvent control. Mitotic cells (M) were collected 11 h after release from thymidine arrest. All mitotic time points were collected by mitotic shake-off (except in Figs. 3b, c and 4a, b where cells were collected by trypsinization).

### Single-cell fate experiments

A total of 2 × 10^4^HeLa cells were seeded into 24-well plates (Ibidi) and transfected with 40 nM siRNA against luciferase (GL2, CGUACGCGGAAUACUUCGATT) or Drp1 (GGAGCCAGCUAGAUAUUAA) using Oligofectamine (Invitrogen) according to the manufacturers instructions 48 h before the start of the imaging. After 18 h, 2 mM of Thymidine (Sigma) was added for 24 h to synchronize the cells. Afterwards the cells were washed twice with PBS and fresh medium with the respective drugs (DMSO (Sigma), 0.5 μM PTX (Sigma) or 1 μM Noc (Sigma)) was added. Two positions per well were imaged every 5 min by a Leica DMi8 microscope with a ×10 objective and a Hamamatsu OrcaFlash 4.0 camera. For the analysis, cells (first cell division of 60 cells for DMSO treatment, all cells for the mitotic inhibitor treatments) entering mitosis were manually followed by using Fiji. The rounding up of the cells was judged as the beginning of mitosis. Mitotic duration was then calculated by determining the time point of anaphase (elongation of the cell shortly before cell division), mitotic death (blebbing and shrinkage of the cell), or mitotic slippage (flattening of the cells) (see also Supplementary Fig. 5).

### TMRE staining

Cells were harvested at indicated time points and stained with 100 nM TMRE in DMEM at 37 °C for 30 min. Cells were washed with PBS and mitochondrial potential was measured by flow cytometry using CytoFlex (Beckman Coulter). Data were analyzed by using the FACSDiva software (Beckman Coulter).

### Annexin V staining

Cells were harvested at indicated time points, washed in PBS and resuspended in 1× Annexin binding buffer (BD Bioscience) containing Annexin V-FITC. After incubation for 15 min in the dark at room temperature, Annexin V-positive cells were measured by flow cytometry using CytoFlex (Beckman Coulter). Data were analyzed by using the FACSDiva software (Beckman Coulter).

### Histone-3 P immunostaining

Attached and nonattached thymidine-arrested cells were collected after 12 h (M), 16 h (M + 4), and 20 h (M + 8) of release in PTX, Noc, or DMSO. Cells were fixed in ice-cold 70% ethanol overnight at −20 °C. After fixation, cells were washed with PBS and dilution buffer (1% FCS, 1% Triton in PBS) and incubated in anti-phosphoH3-Alexa488 (1:200 in dilution buffer) (Cell Signaling) for 1 h at room temperature. Cells were washed in PBS and stained with PI (2.5 µg/mL) for 15 min at room temperature. The DNA content and mitotic population were measured by flow cytometry using CytoFlex and data were analyzed using the FACSDiva software (Beckman Coulter).

### Apoptosis activity assay

For caspase-3/7 measurements, cells were seeded in white bottom 96-well plates (Greiner Bio-One). Cells were left untreated or treated with STS (1 µM, 3 h) or PTX (50 nM, 37 h). Following treatment, cells were subjected to caspase-3/7 activity measurement with Caspase Glo assay kit (Promega). Briefly, equal amounts of Caspase Glo reagent were added to each well and the plate was incubated for 30 min at room temperature. The luminescence of each sample was measured in a plate reader (Infinite M200, Tecan).

### Subcellular fractionation and Bax oligomerization

Subcellular fractionation was performed as described in ref. [55]. Briefly, untreated or STS/PTX-treated HeLa cells were harvested in isotonic mitochondrial buffer (MB: 210 mM mannitol, 70 mM sucrose, 1 mM EDTA, and 10 mM Hepes, pH 7.5) supplemented with protease inhibitor Cocktail Complete (Sigma-Aldrich) and homogenized for 50 strokes with a Dounce homogenizer. Cytosolic and HM fractions were separated by differential centrifugation steps. First, nuclei and unbroken cells were eliminated by sequential centrifugations at 500 g for 5 min at 4 °C. Next, the resulting supernatant was centrifuged at 10,000 × *g* for 30 min at 4 °C to collect HM fractions. To obtain the cytosolic (C) fraction, the resulting supernatant was centrifuged at 100,000 × *g* for 1 h at 4 °C. HM and cytosolic fractions were loaded in a 12% polyacrylamide gel and transferred onto a PDVF membrane (Merck Millipore). After blocking in 5% nonfat milk in TBS-T, incubation with anti-Bax (1:1000, Cell Signaling) was used to assess Bax localization. Anti-VDAC1 (1:1000, abcam), and anti-GAPDH (1:5000, abcam) were used as mitochondrial and cytosolic markers, respectively.

To study Bax oligomerization, HM fractions were incubated with 0.1 mM of BMH crosslinker (Thermo Fisher Scientific) for 45 min at room temperature with gentle agitation. The crosslinker was quenched with 0.5 M DTT (Roth) in MB buffer for 15 min at room temperature. HM fractions were collected by centrifugation at 10,000 × g for 10 min at 4 °C and they were washed twice with MB buffer. After washes, HM fractions were resuspended in 1× sample buffer without DTT and boiled for 5 min at 95 °C. Samples were subjected to western blot as described below.

### Western blotting

Cells were lysed in 50 mM Tris pH 8.0, 150 mM NaCl, 0.5% NP-40 supplemented with protease inhibitors cocktail (Roche). 50 μg of protein were loaded on 10, 12, or 14% polyacrylamide gels and transferred onto PDVF membrane (Merck Millipore) or Amersham Hybond-ECL nitrocellulose membranes (GE Healthcare) using the Turboblot (BioRad) or a wet transfer system (BioRad). Blots were incubated overnight at 4 °C with primary antibodies, probed with secondary antibodies (Jackson ImmunoResearch) and develop using ECL (Perkin Elmer). The following primary antibodies were used: anti-Dlp1 (1:1000, BD Bioscience), anti-VDAC1 (1:1000, abcam), anti-Bax (1:1000, Cell Signaling), anti-actin (1:10.000, Cell Signaling), anti-GAPDH (1:5000, Abcam), anti-PARP (1:1000, Cell Signaling), anti-cleaved caspase-3 (1:1000, Cell Signaling), anti-Mcl1 (1:500, Santa Cruz), anti-Noxa (1:400, Merck), anti-Bcl-2 (1:1000, clone 100), anti-BclX (1:1000, Cell Signaling), anti-Bid (1:1000, clone 8C3, gift from David Huang), anti-Bak (1:1000, Cell Signaling), anti-Mfn2 (1:1000, Cell Signaling).

### Confocal microscope

For Drp1 knockdown experiments, 24 h after siRNA transfection as described above, cells were seeded in eight-well chambers (Ibidi). After cell cycle synchronization, cells were released in phenol red free DMEM (Sigma-Aldrich) supplemented with FBS and P/S containing different drugs. MitoTracker Red CMXRos (Thermo Fisher Scientific) and DAPI (Thermo Fischer Scientific) were directly added to the wells and incubated for 30 min at 37 °C. Images were acquired with a Zeiss LSM 710 ConfoCor3 microscope (Carl Zeiss) equipped with incubator at 37 °C and 5% CO_2_. Transmitted light and fluorescence images were acquired through a Zeiss C-Apochromat 40×, numerical aperture (NA) = 1.2 water immersion objective onto the sample. For experiments testing the role of mitochondrial shape, cells were incubated for 30 min in DMEM containing 200 nM TMRE. Cells were then washed once with PBS and incubated in phenol red free L15 Medium (Invitrogen). Images were taken with a Leica DMi8 microscope with a 63× water objective (NA = 1.2) and a Hamamatsu OrcaFlash 4.0 camera.

### WIPI2 immunostaining

Cells were seeded in six-well plate and siRNA transfection was performed the next day. After 24 h, cells were transferred to eight-well NUNC labtek (Sigma-Aldrich) and cell cycle synchronization using double-thymidine block was performed as described above. Cells were stained with MitoTracker Red CMXROS (100 nM, 30 min at 37 °C) (Thermo Fisher Scientific) at different time points after release from cell cycle arrest. Cells were fixed in 4% PFA for 30 min at room temperature. After blocking for 1 h at 4 °C in 1% blocking buffer (1% BSA, 0.1% Tween20 in PBS), cells were incubated with anti-WIPI2 (1:50, Abgent) for 1 h at 4 °C. Cells were washed twice with PBST and stained with secondary antibody (antirabbit Alexa633, 1:200, Life Technologies) for 1 h at 4 °C. Cells were washed twice with PBS and mounted with ProLong Gold Antifade Mountant (Thermo Fisher Scientific). Transmitted light and fluorescence images were acquired through a Zeiss C-Apochromat 63×, NA = 1.4 oil immersion objective onto the sample. Samples were analyzed using FIJI.

### mKeima experiment

Cells were seeded in six-well plate and siRNA transfection was performed the next day. Cell cycle synchronization using double-thymidine block was performed as described above. Sixty hours after siRNA transfection, mKeima-Red-mito-7 (addgene plasmid #56018) was transfected into the cells using Lipofectamine 2000 (Life Technologies). Cells were harvested at different times after cell cycle arrest release by mitotic shake-off and analyzed by flow cytometry using CytoFlex (Beckman Coulter). Measurements of lysosomal mKeima-Red-mito were made using measurements at 488 and 561 nm lasers with 690/50 emission filters. Data were analyzed using FACSDiva software (Beckman Coulter).

### SILAC experiment

HeLa cells were cultured in media with medium (STS condition) or heavy (PTX condition) isotopes of arginine and lysine for at least six passages. Control cells without treatment were cultured in light media. Incorporation rates of labeled isotopes were at least 98%. Respective treatments were performed in the cultured cells and cells were harvested and stained with TMRE as described above. Cell sorting of cells with at least 50% TMRE intensity was carried out in a BD FACS Aria I (BD Bioscience) in order to discard the dead cells for the mass spectrometry analysis.

Protein extracts derived from the ‘light’, ‘medium,’ and ‘heavy’ labeled cell cultures were mixed in a 1:1:1 ratio according to measured protein amounts. Seventy-five milligrams of the mixture was resolved by SDS–PAGE, and the gel lane was cut into 15 slices, and digested with trypsin as described previously [56]. Extracted peptides were desalted using C18 StageTips [57].

LC-MS/MS analyses of the peptide fractions were performed on an EasyLC nano-HPLC (Thermo Fisher Scientific) system coupled to an Orbitrap Elite (Thermo Fisher Scientific) mass spectrometer as already described [58]. In brief: peptides were eluted from the column using a segmented gradient of 5–50% of solvent B (80% ACN in 0.5% acetic acid) at a constant flow rate of 200 nL/min over 87 min. The 15 most intense precursor ions were sequentially fragmented in each scan cycle. High-resolution HCD MS/MS spectra were acquired with a resolution of 15,000.

The target values for the MS scan and MS/MS fragmentation were 106 and 40,000 charges, respectively. Precursor ions were excluded from sequencing for 60 s after MS/MS.

### MS data processing

The acquired MS raw files were processed using default parameters of the MaxQuant software (v1.5.2.8) [59]. Extracted peak lists were submitted to database search using the Andromeda search engine [60] to query a target-decoy [61] database consisting of the homo sapiens proteome (88,692 entries), and commonly observed contaminants (248 entries). Full tryptic specificity was required allowing up to two missed cleavages and set the minimal peptide length to seven amino acids. The initial precursor mass tolerance was set to 4.5 ppm, for fragment ions we used a mass tolerance of 20 ppm. For reduced and alkylated samples carbamidomethylation of cysteines was defined as fixed modification in the database search. Protein N-terminal acetylation, and oxidation of methionine were set as variable modifications. Peptide, protein and modification site identifications were filtered at a false discovery rate set to 0.01. For protein group quantitation a minimum of two quantified peptides were required.

Perseus software (version 1.5.0.15), a module from the MaxQuant suite, was used for calculation of the significance B (psigB) for each protein ratio with respect to the distance of the median of the distribution of all protein ratios as well as its intensities. All proteins with psigB < 0.01 in a pairwise comparison were considered to be differentially expressed.

To identify those proteins involved in apoptosis, all the resulting proteins of the SILAC experiment were loaded into PHANTER DB [62] to obtain their correspondent GOBP annotation. Proteins that contain the term ‘apopt’ in their GOBP were classified as apoptotic proteins. A heat map was plotted for those proteins involved in apoptosis using the Log_2_ ratio of each condition comparison.

### Generation of Mfn2 overexpressing cell lines

Human Mfn2 cDNA was cloned into an MSCV IRES puromycin (pMIP) vector. HeLa cells were transduced with either pMIP (empty vector) or pMIP containing hMfn2. Cells were then selected with 2 µg/mL puromycin (Sigma-Aldrich) for 5 days.

## Supplementary information

Supplementary material

Supplementary figure 1

Supplementary figure 4

Supplementary figure 5

Supplementary figure 6

Supplementary figure 7

Supplementary figure 8

Supplementary figure 2

Supplementary figure 3
