# Correction: Drp1 modulates mitochondrial stress responses to mitotic arrest

**DOI:** 10.1038/s41418-021-00831-x

**Published:** 2021-07-23

**Authors:** Aida Peña-Blanco, Manuel D. Haschka, Andreas Jenner, Theresia Zuleger, Tassula Proikas-Cezanne, Andreas Villunger, Ana J. García-Sáez

**Affiliations:** 1 Interfaculty Institute of Biochemistry, Eberhard Karls University Tübingen, Tübingen, Germany; 2 Division of Developmental Immunology, Biocenter, Medical University of Innsbruck, Innsbruck, Austria; 3 Department of Molecular Biology, Interfaculty Institute of Cell Biology, Eberhard Karls University Tübingen, Tübingen, Germany; 4 Ludwig Boltzmann Institute for Rare and Undiagnosed Diseases, 1090 Vienna, Austria; 5 CeMM Research Center for Molecular Medicine of the Austrian Academy of Sciences, 1090 Vienna, Austria; 6 Present Address: Institute of Genetics, CECAD, University of Cologne, Cologne, Germany

**Keywords:** Cell biology, Cell death and immune response

Correction to: *Cell Death & Differentiation* 10.1038/s41418-020-0527-y

The article Drp1 modulates mitochondrial stress responses to mitotic arrest, written by Aida Peña-Blanco, Manuel D. Haschka, Andreas Jenner, Theresia Zuleger,Tassula Proikas-Cezanne, Andreas Villunger and Ana J. García-Sáez, was originally published electronically on the publisher’s internet portal on 19 March 2020 without open access. With the author(s)’ decision to opt for Open Choice the copyright of the article changed on 5 July 2021 to © The Author(s) 2021 and the article is forthwith distributed under a Creative Commons Attribution 4.0 International License, which permits use, sharing, adaptation, distribution and reproduction in any medium or format, as long as you give appropriate credit to the original author(s) and the source, provide a link to the Creative Commons licence, and indicate if changes were made. The images or other third party material in this article are included in the article’s Creative Commons licence, unless indicated otherwise in a credit line to the material. If material is not included in the article’s Creative Commons licence and your intended use is not permitted by statutory regulation or exceeds the permitted use, you will need to obtain permission directly from the copyright holder. To view a copy of this licence, visit http://creativecommons.org/licenses/by/4.0/.

## Funding

Open Access funding enabled and organized by Projekt DEAL.

