# Supplementary material for: Drp1 modulates mitochondrial stress responses to mitotic arrest
Source: Cell Death Differ. 2020 Mar 19;27(9):2620–34. doi: 10.1038/s41418-020-0527-y (PMC7429963; doi:10.1038/s41418-020-0527-y)
Supplement: Supplementary file 1 — Supplementary material [file 41418_2020_527_MOESM1_ESM.pdf]

## Supplementary figures

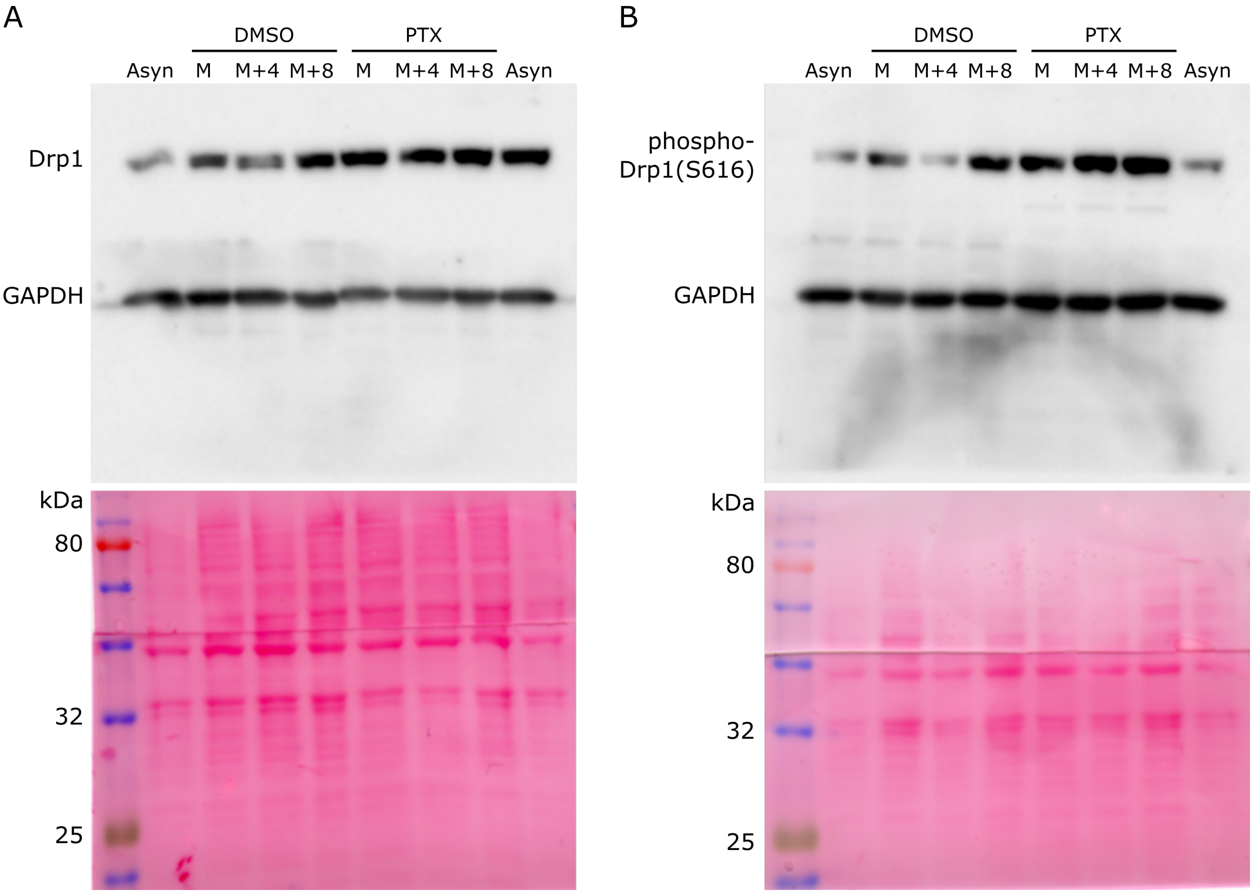


**Figure S1. Accumulation of Drp1 and phosphorylated Drp1 during mitotic arrest validated by western blot.**

Immunoblot for A) Drp1 and B) phosphorylated Drp1 (Drp1-pS616) in HeLa cells released in DMSO or PTX after thymidine blockage. Cells were harvested in M phase or after 4-8 h of mitotic arrest (M+4, M+8). Immunostaining of GAPDH and Ponceau S staining (lower panels) was used as loading control. Asyn, asynchronous cells

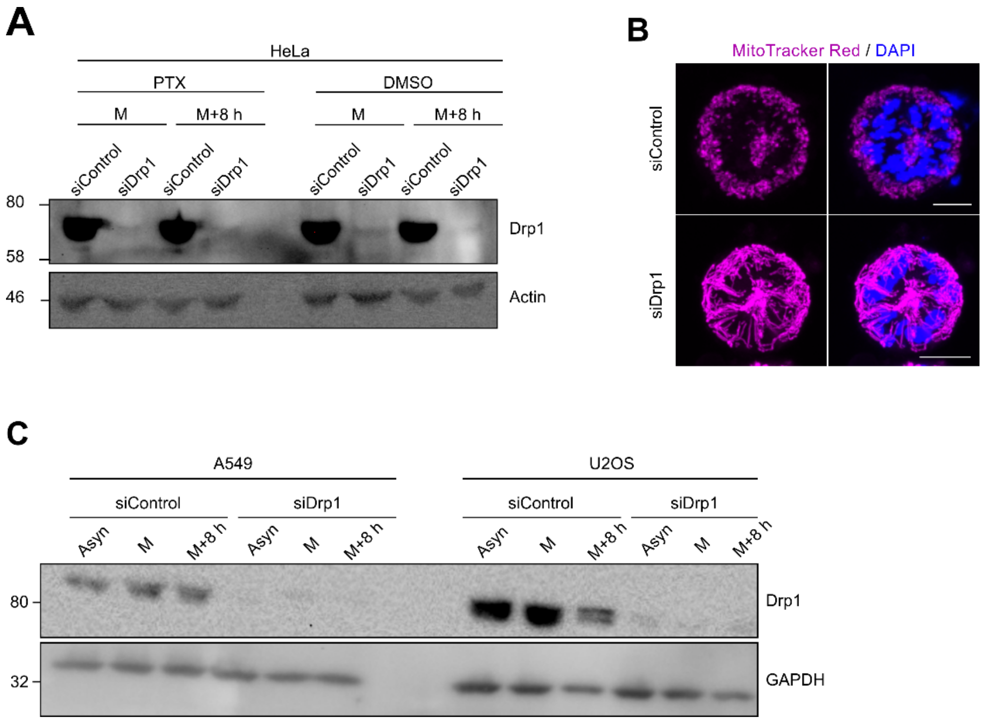


**Figure S2. DRP1 levels are decreased in siRNA-DRP1 cells.**

A) Immunoblot for DRP1 in siControl and siDRP1 HeLa cells released into PTX or DMSO after double thymidine block. Actin was used as a loading control. B) Representative images of mitotic siControl and siDRP1 HeLa cells. Mitochondria and DNA were stained with Mitotracker Red CMXROs and DAPI, respectively. Scale bar, 10 µm. C) Immunoblot for DRP1 in siControl and siDRP1 U2OS and A549 cells released into PTX or DMSO after double thymidine block. Asyn, asynchronous cells. GAPDH was used as a loading control.

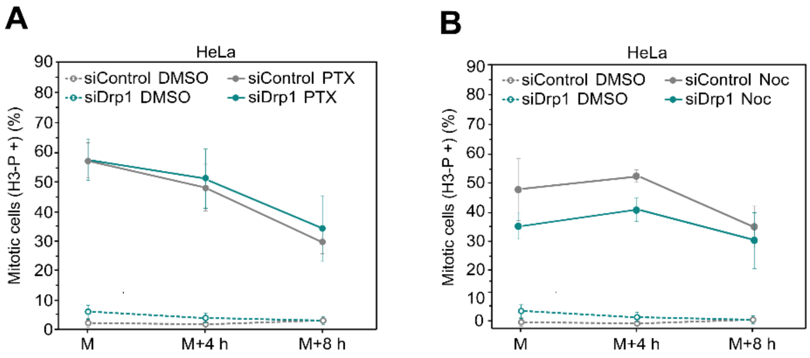


**Figure S3. DRP1 downregulation does not affect mitotic population in HeLa cells.** Quantification of the percentage of mitotic cells in siControl (grey) and siDRP1 (blue) HeLa cells released into media with PTX (A) or Noc (B). DMSO-released cells (empty circles) are shown as a control. The values represent the mean and the SD of four independent experiments.

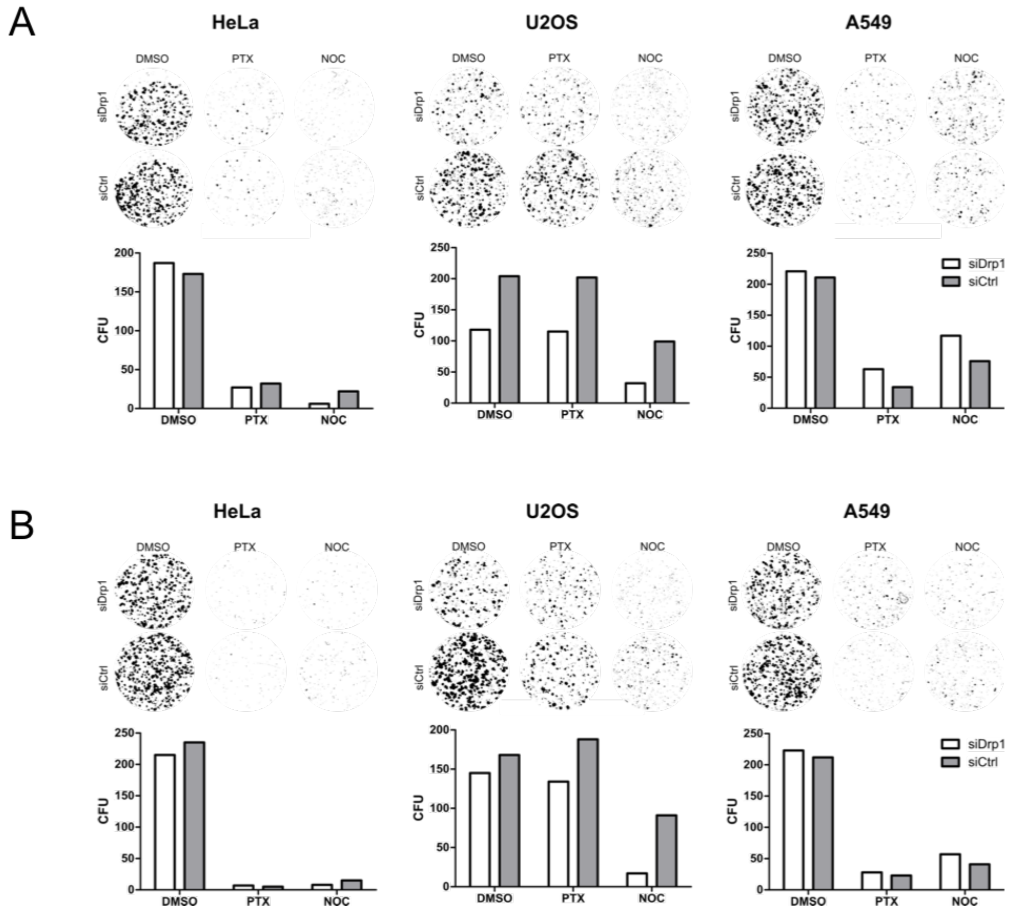


**Figure S4. Colony formation assay for the indicated cell lines treated with DMSO, taxol (PTX) or nocodazole, and with siDrp1 or a control siRNA (siCrtl). A) M, B) M+4.** We compared the growth potential of wild type cells with those treated with Drp1 siRNA in steady state and and upon transient mitotic arrest induced by PTX or Noc treated for 12h (M) or 16h (M+4) followed by washout of the drug. Very few HeLa cells survived drug treatment and were able to form colonies. In A549 cells and U2OS cells, that are more slippage prone, more cells survived drug-treatment and formed colonies, but no substantial difference was observed between wt and siDrp1 treated cells.

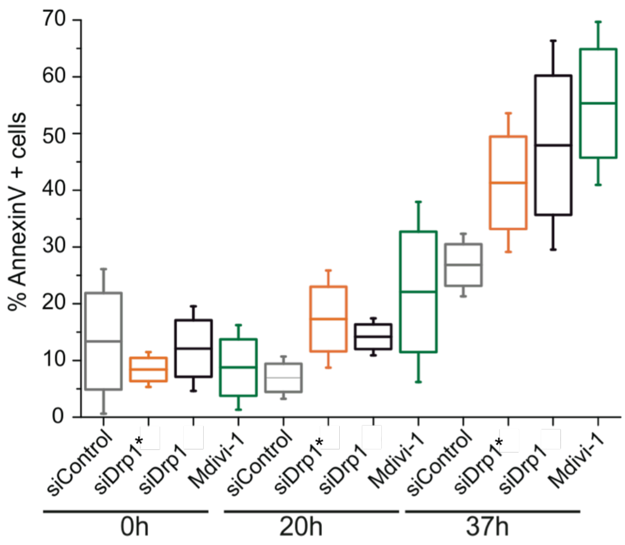


**Figure S5. The effect of DRP1 depletion on cell death measured by PS exposure is reproduced with an alternative siRNA and with Mdivi-1 treatment.** In addition to the siRNA used throughout the study, we confirmed in HeLa cells that the effect of DRP1 depletion on PS exposure and AnnexinV binding was similar when a different siRNA was used (siDrp1*, with sequence GAAAGAAGCAGCUGAUAUG) or with Mdivi-1 treatment.

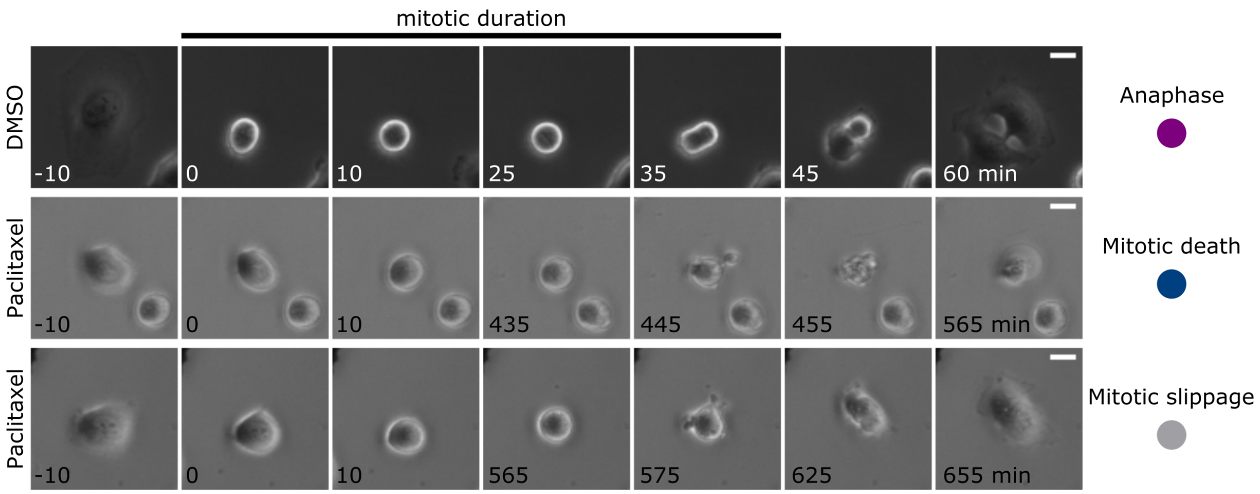


**Figure S6. Representative images of HeLa cells transfected with control siRNA and treated with either DMSO or Paclitaxel**. The upper black bar indicates the assessed mitotic duration beginning from the rounding up of the cells to either anaphase (indicated by elongation of the cell followed by cytokinesis, purple color), mitotic death (blebbing and shrinkage of the cell, blue color) or mitotic slippage (flattening of the cell, grey color). Time in minutes is indicated. Scale Bar is 10 µm.

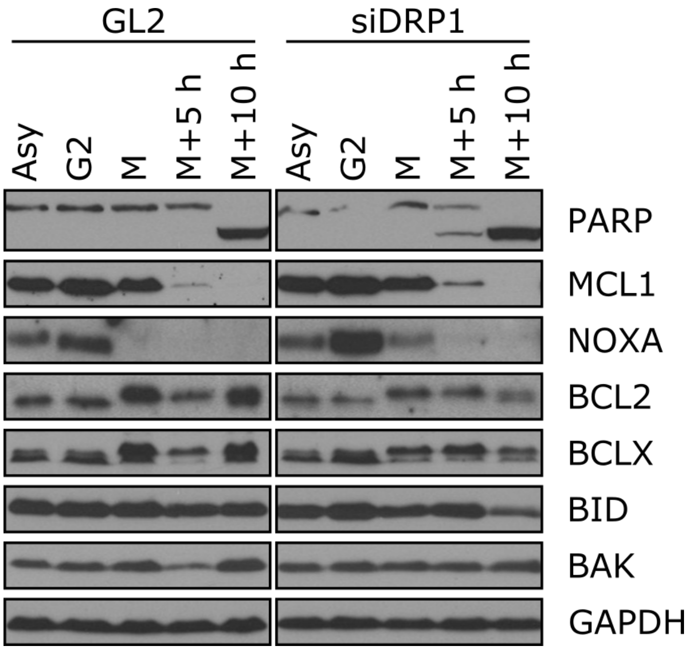


**Figure S7. PARP cleavage is enhanced in siDRP1 HeLa cells during mitotic arrest.** Asynchronous (Asy) HeLa cells or HeLa cells synchronized by double-thymidine block and released into PTX-containing media were harvested in G2 and early M phase or after 5-10 h of mitotic arrest. Cells were harvested, lysed and analysed by immunoblotting.

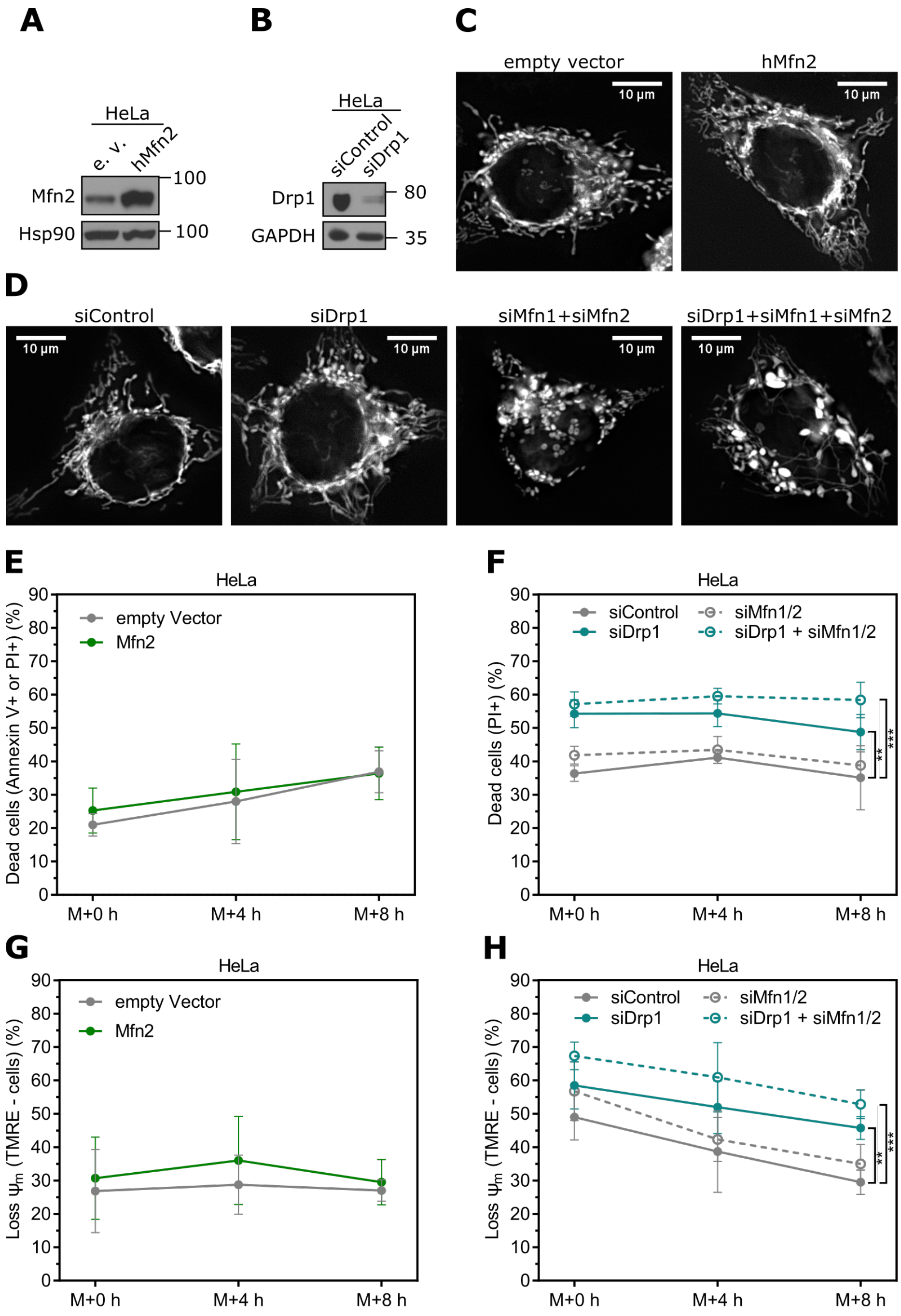


**Figure S8. Role of mitochondrial shape on Drp1 effects on mitotic cell death.** A) Mfn2 immunoblot of HeLa cells transduced with either an empty vector (e. v.) or an expression vector for human Mfn2 (hMfn2). B) Drp1 immunoblot of HeLa cells transduced with an empty vector and transfected with either control siRNA or siRNA against Drp1. C) TMRE stained mitochondria of HeLa cells transduced with either empty vector or human Mfn2 over expression. D) TMRE stained mitochondria of HeLa cells transfected with the indicated siRNAs. E) Quantification of the percentage of HeLa cells with either PS exposure at the plasma membrane or uptake of propidium

iodide (PI) in cells transduced with either empty vector (grey) or a vector expressing human Mfn2 (green) released into Media with PTX. Mitotic cells were harvested 12 h (M+0 h), 16 h (M+4 h) and 20 h (M+8 h) after release from a double thymidine block by mitotic shake off. The values represent the mean and the SD of three independent experiments. F) Quantification of the percentage of HeLa cells with uptake of propidium iodide (PI) in cells transfected with the indicated siRNAs released into Media with PTX. Mitotic cells were harvested 12 h (M+0 h), 16 h (M+4 h) and 20 h (M+8 h) after release from a double thymidine block by mitotic shake off. The values represent the mean and the SD of three independent experiments. *** $p < 0.001$, ** $p < 0.01$ using two-way ANOVA and Tukey's multiple comparisons test. G) Same as E), but instead of PS exposure and PI uptake $\Psi$m loss was measured. H) Same as F), but instead of PI uptake $\Psi$m loss was measured. *** $p < 0.001$, ** $p < 0.01$ using two-way ANOVA and Tukey's multiple comparisons test.
