# Supplementary figures and images for: Drp1 modulates mitochondrial stress responses to mitotic arrest
Source: Cell Death Differ. 2020 Mar 19;27(9):2620–34. doi: 10.1038/s41418-020-0527-y (PMC7429963; doi:10.1038/s41418-020-0527-y)

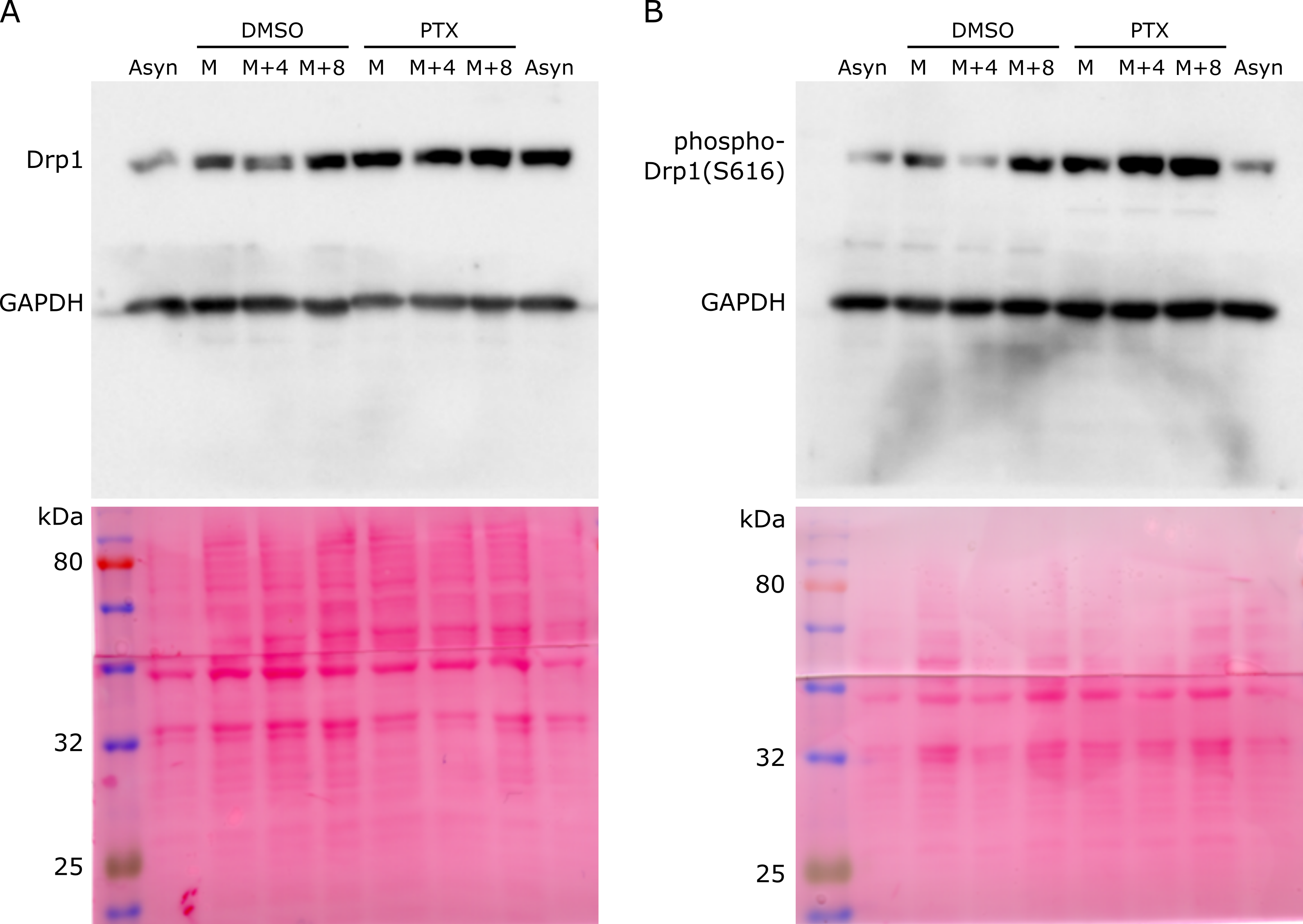

Supplement: Supplementary file 2 — Supplementary figure 1 [file 41418_2020_527_MOESM2_ESM.png]

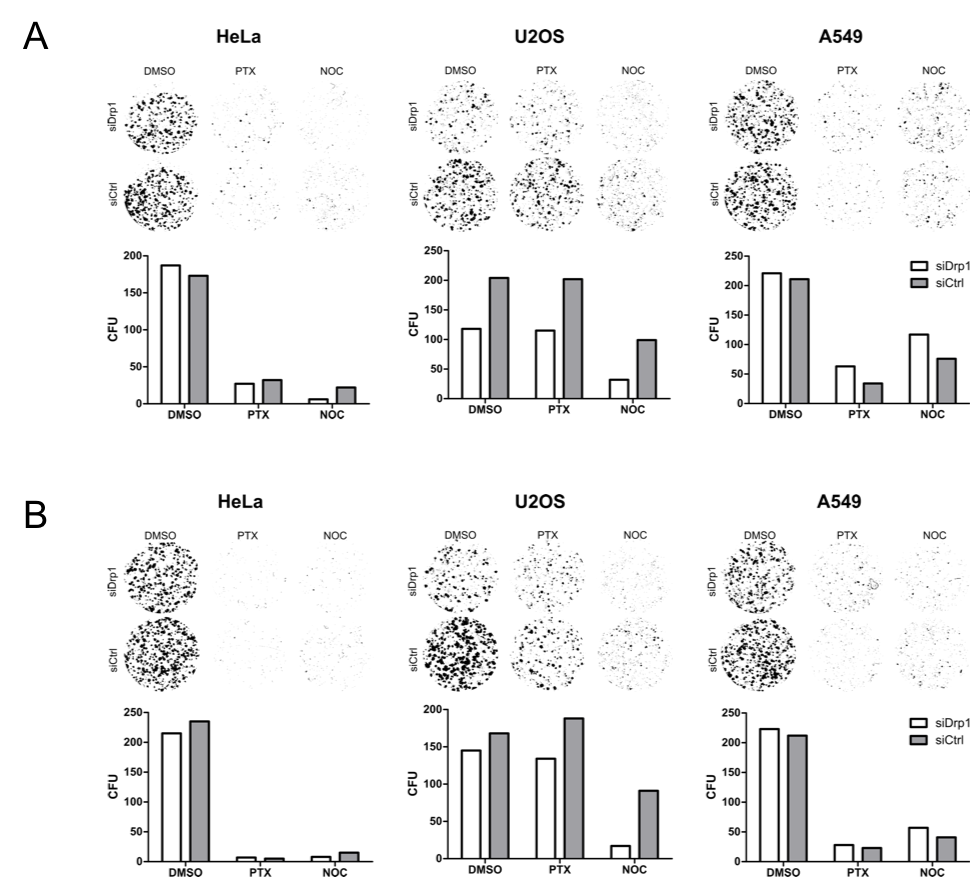

Supplement: Supplementary file 3 — Supplementary figure 4 [file 41418_2020_527_MOESM3_ESM.png]

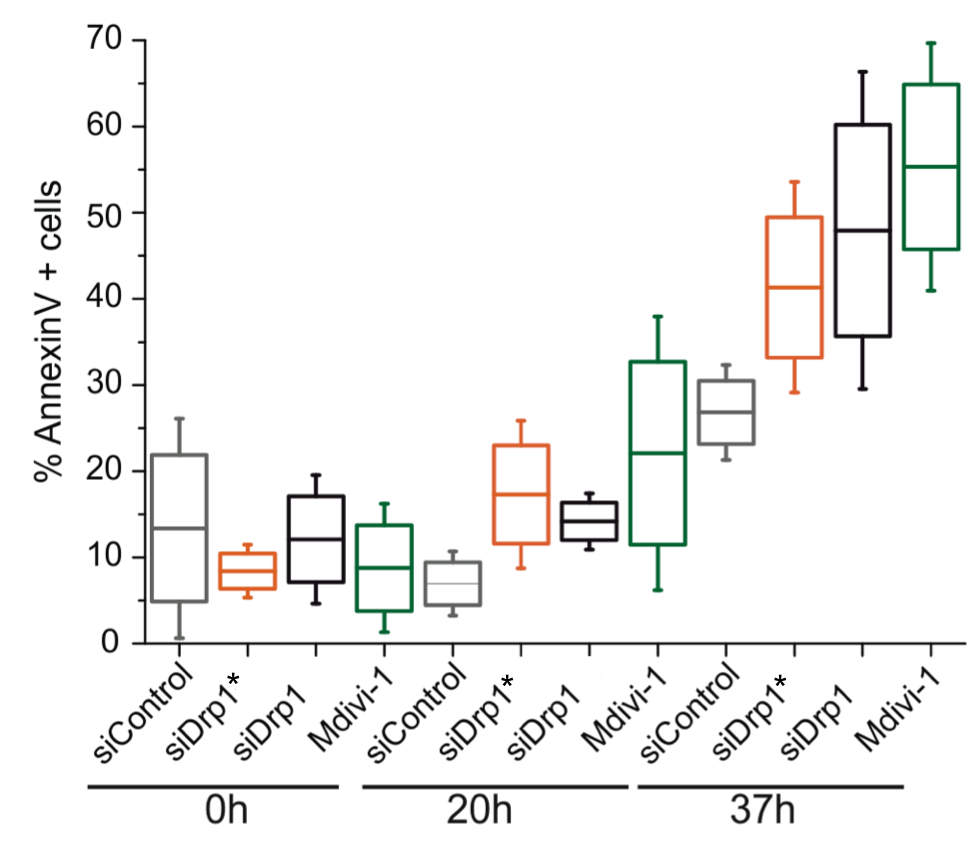

Supplement: Supplementary file 4 — Supplementary figure 5 [file 41418_2020_527_MOESM4_ESM.png]

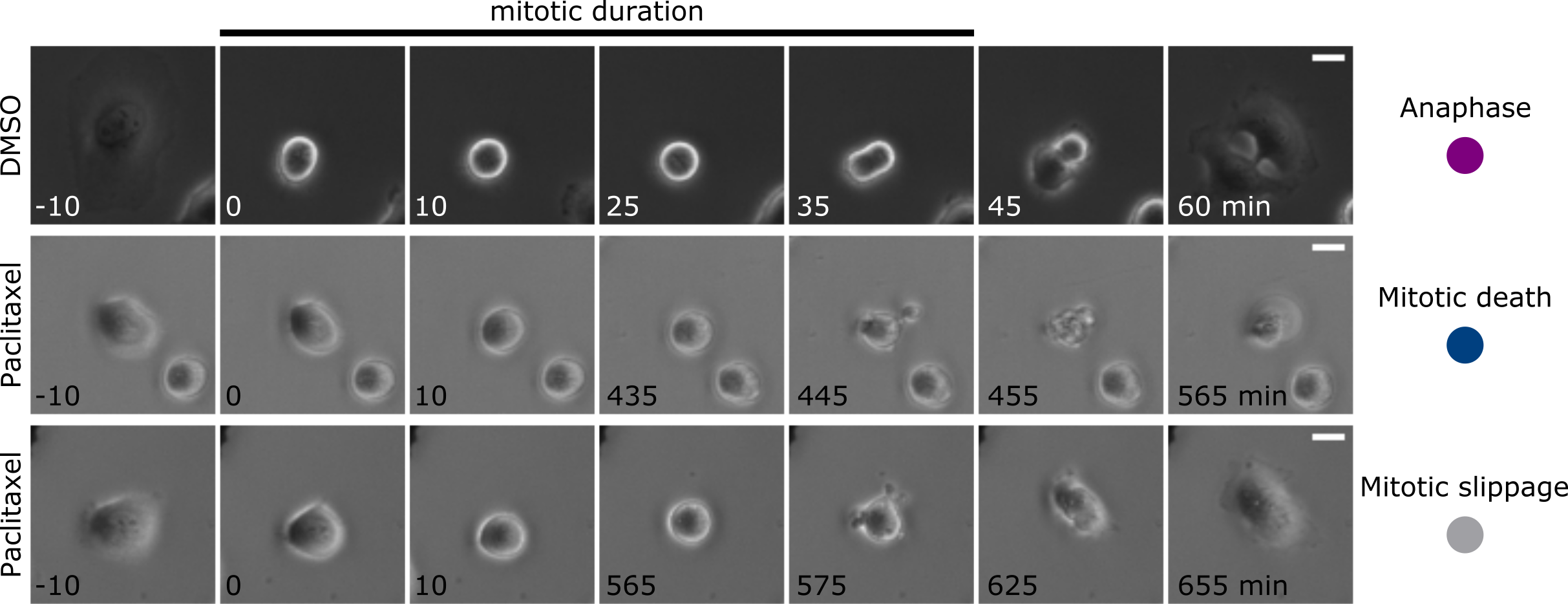

Supplement: Supplementary file 5 — Supplementary figure 6 [file 41418_2020_527_MOESM5_ESM.png]

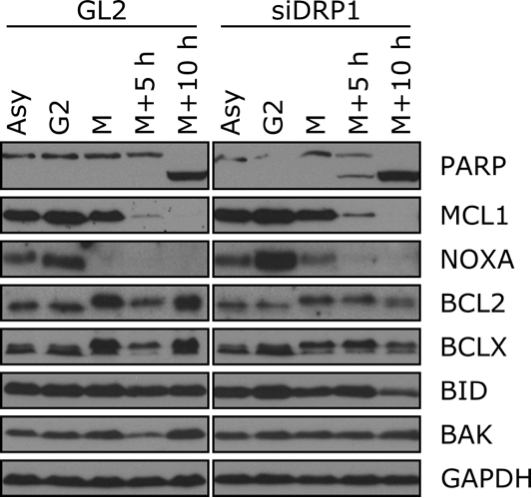

Supplement: Supplementary file 6 — Supplementary figure 7 [file 41418_2020_527_MOESM6_ESM.png]

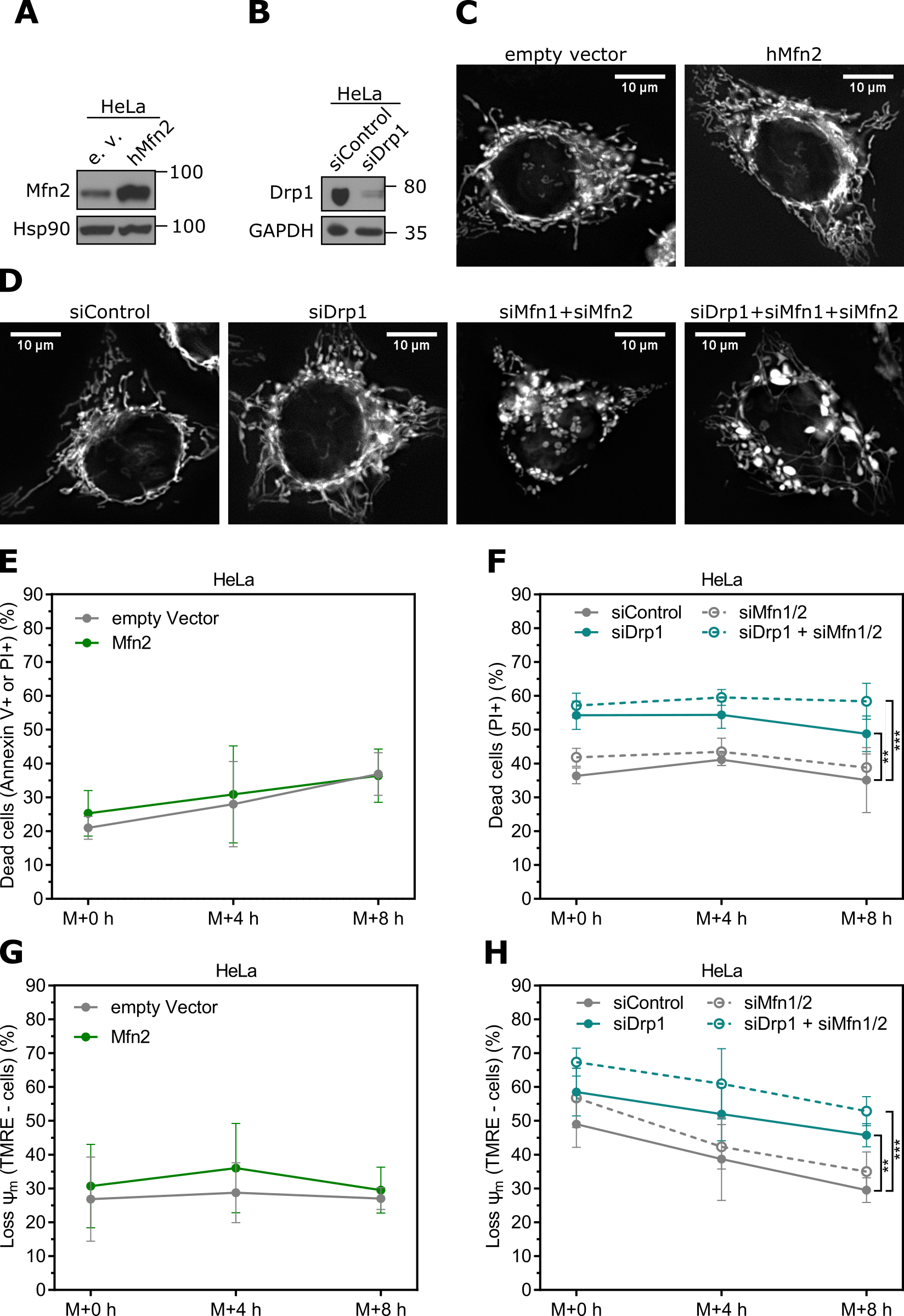

Supplement: Supplementary file 7 — Supplementary figure 8 [file 41418_2020_527_MOESM7_ESM.png]

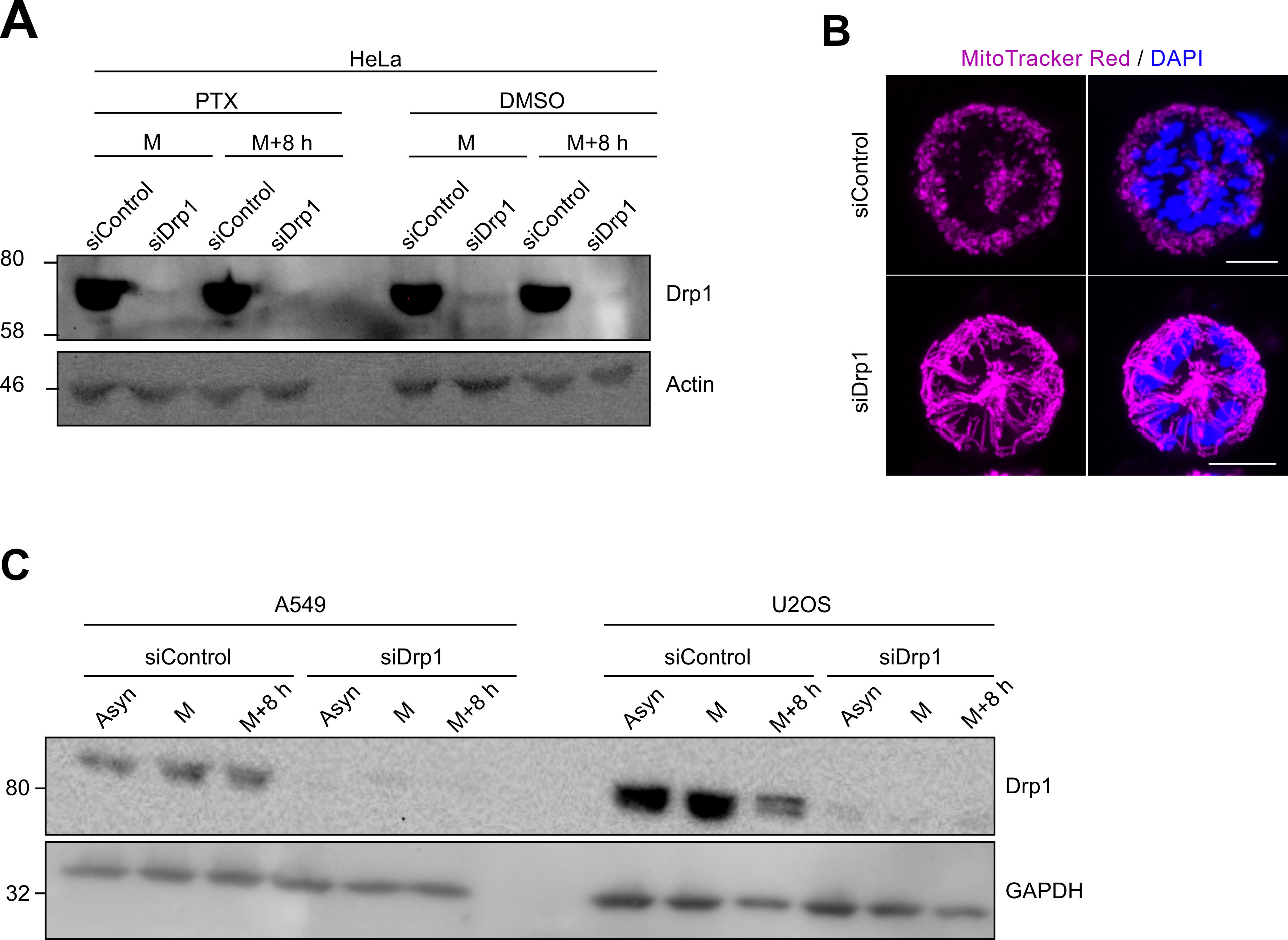

Supplement: Supplementary file 8 — Supplementary figure 2 [file 41418_2020_527_MOESM8_ESM.tif]

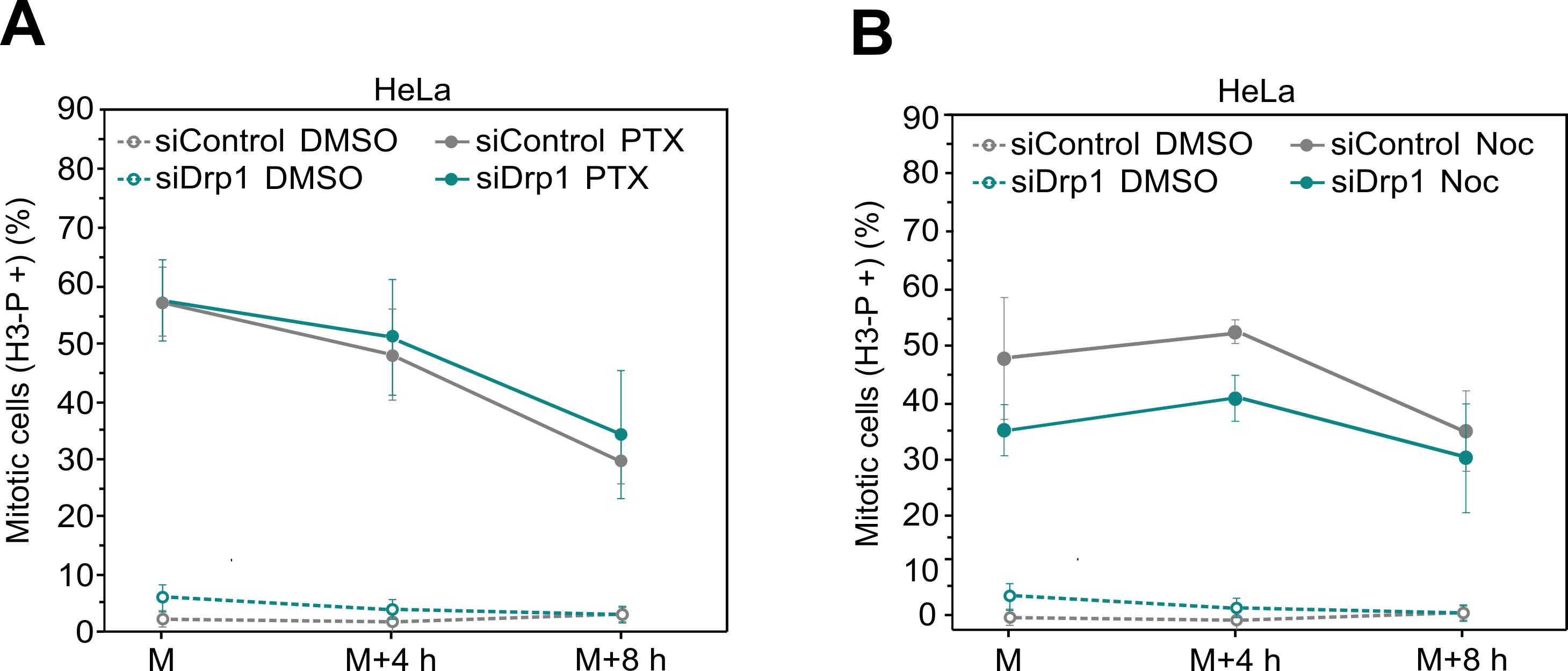

Supplement: Supplementary file 9 — Supplementary figure 3 [file 41418_2020_527_MOESM9_ESM.tif]
